# Identification of stemness-related glycosylation changes in head and neck squamous cell carcinoma

**DOI:** 10.1186/s12885-024-12161-5

**Published:** 2024-04-10

**Authors:** E Routila, R Mahran, S Salminen, H Irjala, E Haapio, E Kytö, S Ventelä, K Petterson, J Routila, K Gidwani, J Leivo

**Affiliations:** 1 Department of Life Technologies, University of Turku, Kiinamyllynkatu 10, 20520 Turku, Finland; 2 InFLAMES Research Flagship, University of Turku, 20014 Turku, Finland; 3 FICAN West Cancer Centre, Turku, Finland; 4 Department of Chemistry, University of Turku, Henrikinkatu 2, 20500 Turku, Finland; 5 Department for Otorhinolaryngology– Head and Neck surgery, University of Turku and Turku University Hospital, Savitehtaankatu 5, 20520 Turku, Finland; 6 Turku Bioscience Centre, University of Turku and Åbo Akademi University, Tykistökatu 6, 20520 Turku, Finland

**Keywords:** HNSCC, Stemness, Glycosylation, Lectin, Bioaffinity assay

## Abstract

**Background:**

Altered glycosylation is a hallmark of cancer associated with therapy resistance and tumor behavior. In this study, we investigated the glycosylation profile of stemness-related proteins OCT4, CIP2A, MET, and LIMA1 in HNSCC tumors.

**Methods:**

Tumor, adjacent normal tissue, and blood samples of 25 patients were collected together with clinical details. After tissue processing, lectin-based glycovariant screens were performed.

**Results:**

Strong correlation between glycosylation profiles of all four stemness-related proteins was observed in tumor tissue, whereas glycosylation in tumor tissue, adjacent normal tissue, and serum was differential.

**Conclusions:**

A mannose- and galactose-rich glycosylation niche associated with stemness-related proteins was identified.

**Supplementary Information:**

The online version contains supplementary material available at 10.1186/s12885-024-12161-5.

## Background

Head and neck cancer is the 7th most common malignancy across the globe with almost 700,000 diagnosed cases annually. The unimproved 5-year survival rate of approximately 50% leads to annual death toll of over 300,000. Over 90% of head and neck cancer are squamous cell carcinomas (HNSCC). The incidence rates of HNSCC are on the rise, due to population aging and the ongoing human papilloma virus (HPV) epidemic [1, 2], whereas the traditional risk factors associated with mutagenic exposure of the mucosal lining of the upper aerodigestive tract still play an important role both as risk factors and markers for adverse survival of HNSCC [3].

The HNSCC tumors express a wide range of stemness-associated biomarkers such as OCT4 [4, 5], and MET [6]. Stemness has a well-established role in radioresistance of HNSCC and several stemness-related biomarkers have been suggested. MET was previously shown to be an adverse prognostic indicator in non-metastatic HNSCC [7], and OCT4 has been associated with radiotherapy and chemotherapy resistance [8, 9]. Importantly, however, the negative prognostic impact of OCT4 positivity may be reversed with cisplatin [8]. Due to the heterogeneous nature of HNSCC, the therapeutic implications of such increased expression have been slow to emerge. However, in our previous studies, we have demonstrated that OCT4 and CIP2A play a significant role with regard to radiosensitivity, the most crucial problem in the therapy planning of advanced HNSCC [4, 5, 8].

Altered glycosylation regulates cancer development and progression by affecting tumor growth, invasiveness, and metastasis [10, 11]. A recent study using TCGA datasets suggested that glycosylation-related genes could be used as prognostic risk markers or to guide immunotherapy stratification in HNSCC [12]. The activity of stemness-related proteins is similarly regulated by glycosylations [13, 14] and the resulting protein glycovariants are an emerging group of cancer-related biomarker candidates. As previously described by our group [15–19], a novel type of sandwich bioaffinity assay combining the glycan specificity of lectins with the enhanced sensitivity of highly fluorescent europium(III)-nanoparticles enables the detection of specific protein glycovariants directly from patient samples. This method utilizes the avidity effect of multiple glycan-binding lectins per nanoparticle and multiplied fluorescent signal of thousands of europium chelate molecules per nanoparticle to provide low detection limits, allowing for the identification of low-abundant protein glycovariants typically missed by currently used assays.

In this study, glycovariants of stemness-related biomarker candidates OCT4, MET, CIP2A and LIMA1 were screened from two HNSCC cell lines and, further, from a panel of 25 HNSCC patients using samples of tumor tissue, adjacent normal tissue, and serum samples (Supplemental Fig. 1). Novel sandwich bioaffinity assays utilizing lectin-conjugated nanoparticle reporters recognizing six different glycan structures were used. The overall aim was to identify novel stemness-related protein glycovariants and to evaluate their relation to clinical prognostic factors and tumor characteristics. Ultimately, the predictive potential of these new biomarker candidates in therapy stratification was evaluated.

## Methods

### Cell lines

Two previously described patient-derived HNSCC cell lines, UT-SCC-14 and UT-SCC-60B, were used in the study [20]. Cells were cultured in Dulbecco’s Modified Eagle’s Medium (DMEM) with 10% fetal calf serum (FCS), glutamine and antibiotics (penicillin and streptomycin) and were harvested by trypsinization before pelleting the cells. Both the cell lysates and the cell culture media were screened in this study.

### Human HNSCC patient tumor and blood samples

The patient cohort included prospectively recruited patients diagnosed and treated for new HNSCC in Southwestern Finland regional tertiary referral center of Turku University Hospital between 2020 and 2021. Turku University Hospital, due to the unbiased treatment referral system of the Nordic countries, is responsible for treatment of all HNSCC cases diagnosed in Southwestern Finland with a population of 700 000. All patients were treated with curative intent and were alive at the end of the follow-up period, with median follow-up time of 12.7 months. Patient informed consent was obtained, and surgical biopsy samples (*n* = 25) from tumor and adjacent normal tissue were collected in accordance with the local research ethics council permit (Dnro 166/1801/2015). The tumor samples were collected from macroscopically evident tumor center, which was visible on preoperative imaging studies, and later confirmed in postoperative histological analysis. Adjacent normal tissue samples were cut from the resection boundary and histologically confirmed not to contain dysplasia or invasive cancer. Blood samples were collected on the day of surgery. Patient details, including treatment and follow-up data were meticulously recorded.

### Tissue homogenization

The HNSCC and adjacent normal tissue samples were homogenized to release the proteins of interest for lectin assay screening. The tissue samples were kept on ice throughout the homogenization process. The homogenization buffer volume was adjusted so that the disperser blades were immersed in the buffer. Thus, for tissue pieces less than 4 mg, 1 mL RIPA (radioimmunoprecipitation assay buffer, 150 mM NaCl, 50 mM Tris, 5 mg/ml sodium deoxycholate, 1 mg/ml sodium dodecyl sulphate, 1% NP-40 (nonionic polyoxyethylene 40)) per 3,25 mg tissue sample was used, for pieces < 15 mg, 1 mL RIPA per 7,5 mg sample was used, and for pieces > 15 mg, 1 mL RIPA per 15 mg sample was used. When the samples were later diluted for lectin assay, the sample dilutions during homogenization were reversed. Also, 10 µl/ml Halt™ Protease and Phosphatase Inhibitor Cocktail (Thermo Fisher Scientific) was added to minimize protein degradation. The tissue pieces were homogenized mechanically with ULTRA-TURRAX® tissue homogenizer (IKA-Werke GmbH & Co. KG) for 30 s and incubated for further 1 h on ice with occasional mixing by vortex. The cell debris was removed by centrifugation at 10 000 g for 15 min at + 4 °C, and the supernatant was stored at -70 °C. The reproducibility of the homogenization process was tested by measuring the total protein concentration of tissue lysates with BCA protein assay kit (Sigma-Aldrich, USA) according to the manufacturer’s instructions.

### Preparation of biotinylated antibodies and lectin-nanoparticles

The capture antibodies of lectin assays were prepared by biotinylating mouse anti-LIMA1 Mab (sc-136,399), anti-OCT4 Mab (sc-5279), anti-MET Mab (sc-8057) and anti-CIP2A Mab (sc80659) (Santa Cruz Biotechnology Inc, US) as described previously [15]. The Mabs were washed three times with 0.9% NaCl in 10 K Amicon Ultra centrifugal filter (Merck KGaA, Darmstadt, Germany), 10 000 g for 5 min, in order to remove Tris and NaN_3_. For biotinylation, 40-fold molar excess of biotin isothiocyanate (BITC) (University of Turku, Finland) in 50 mM NaHCO_3_ (pH 9.8) was used in final volume of 200 µl, for 4 h, RT, dark. The biotinylated Mabs were purified twice by NAP^TM^-5 and NAP^TM^-10 gel filtration columns (GE Healthcare, Schenectady, NY, USA) with 50 mmol/L Tris-HCl (pH 7.75), containing 150 mmol/L NaCl and 0.5 g/L NaN_3_, and finally stored in 1 g/L BSA at + 4 °C. The performance of each new batch of bio-Mabs and lectin-NPs was compared in lectin bioaffinity assays in parallel with old batches to ensure good quality and effectiveness, and reproducibility of results.

The previously reported [21] europium(III)-chelate-doped nanoparticle (Eu-NP) reporter technology was utilized by conjugating glycan-binding lectins or Mabs on Fluoro-Max™ polystyrene nanoparticles (diameter 95 nm) (Seradyn Inc, Indianapolis, IN, USA). A panel of 17 lectins or other glycan-binding proteins (Table 1) with good coverage of different glycan structures was used in the cell line screening to narrow down the most promising ones (*N* = 6) for the following HNSCC tissue and serum sample screening. The lectins were purchased from Vector laboratories (Burlingame, CA, USA) and C192 Mab was provided by Fujirebio Diagnostics AB (Göteborg, Sweden). The carboxyl groups of 1 × 10^12^ Eu-NPs in 50 mM 2-(morpholino)ethanesulfonic acid (MES) buffer (pH 6.0) were activated by 8 mM N-hydroxysulfosuccinimide (NHS) and 1.3 mM 1-ethyl-3-(3’dimethylaminopropyl)carbodiimide (EDC) in final volume of 110 µl, at RT, vortexing for 15 min. 0,5 mg/ml lectins were conjugated on activated Eu-NPs in final volume of 200 µl by vortexing for 30 min, then changing the pH to 8.0 by 0.5 M carbonate buffer and vortexing for another 30 min. The remaining free carboxyl groups were blocked by 1% BSA by vortexing for 30 min. The lectin-NP-conjugates were washed three times with 25 mM Tris, 150mM NaCl, 0.1% NaN_3_ pH 7.8, and stored in 10 mM Tris-HCl (pH 7.8), 1 g/L BSA and 0,1 g/L NaN_3_ at + 4 °C. Prior to every lectin assay, the lectin-NPs were vortexed for 30 s to disperse any aggregates.

**Table 1:** Lectin panel

**Abbreviation**	**Name**	**Lectin source**	**Carbohydrate specificity**
**UEA**	Ulex Europaeus Agglutinin I	*Ulex Europaeus*	α-linked fucose
**DC-SIGN**	Dendritic cell-specific ICAM-3-grabbing non-integrin, CD209	mammalian	mannose, nonsialylated Lewis antigens
**MGL**	macrophage galactose lectin	mammalian	terminal α- or β-linked GalNAc
**MBL**	Mannose-binding lectin	mammalian	mannose
**C192 Mab**	C192	mouse	CA19.9 / Sialyl Lewis a
**WGA**	Wheat germ agglutinin	*Wheat germ*	GlcNAc
**TJA II**	Trichosanthes japonica agglutinin II	*Trichosanthes japonica*	fucose, lactose
**AAL**	Aleuria aurantia lectin	*Aleuria aurantia lectin*	α-1-6-fucose
**CON A**	Concanavalin A	*Canavalia ensiformis*	α-D-mannose, α-D-glucose
**Gal_3**	galectin-3	mammalian	β galactose
**SBA**	Soybean agglutinin	*Glycine max*	terminal α- or β-linked GalNAc, galactose
**HPA**	Helix pomatia agglutinin	*Helix pomatia*	GalNAc
**Jacalin**	Jacalin	*Artocarpus integrifolia*	Galβ (1–3)GalNAc
**VVL**	Vicia Villosa lectin	*Vicia Villosa*	terminal α- or β-linked GalNAc
**RCA**	Ricinus communis agglutinin	*Ricinus communis*	galactose, lactose
**Gal-7**	galectin-7	mammalian	galactose, LacNAc
**MAA**	Maackia amurensis agglutinin	*Maackia amurensis*	Siaα2-3Galβ1-4GlcNAc
**WFL**	Wisteria floribunda lectin	*Wisteria floribunda*	GalNAc

### Nanoparticle-aided lectin bioaffinity assay for glycovariant screening

A modified in-house sandwich-type bioaffinity assay utilizing lectin-NP-tracers as previously described [15] was used to screen the glycovariants of LIMA1, OCT4, MET and CIP2A in HNSCC cell lines, tissue and serum. The Red assay buffer, Wash buffer and Yellow streptavidin-coated 96-well microtiter plates were purchased from Kaivogen Oy (Turku, Finland). 50 ng/25 µl/well biotinylated capture antibodies were bound on streptavidin-coated microtiter plate at RT for 1 h. After two times washing, 1:500 dilution of cell lysate, 1:2 dilution of cell culture media, 1:15 or 1:7,5 or 1:3,25 dilution of tissue lysate or 1:50 dilution of serum was added and incubated at RT, slow shaking, for 1 h. After washing twice, 1 × 10^7^/25µl lectin-coated Eu^3+^-nanoparticles were added and incubated at RT, ss, for 1,5 h. After six times washing, time-resolved fluorescence was measured with Hidex Sense (Hidex Oy, Turku, Finland) (λex = 340 nm and λem = 615 nm).

### Statistical analysis

All cell line data, patient characteristics and follow-up information, and lectin assay results were inserted into SPSS 28 software (SPSS, IBM). Paired t-test was used to evaluate expression levels in tumor versus normal tissue samples and serum samples. For analysis of correlation between expression levels of different glycovariants, correlation was assessed using the Kendall rank correlation coefficient with interpretation of the correlation strength, where tau-b (τ_b_) values ranging from 0.0 to 0.2 are considered very weak, values from 0.2 to 0.4 weak, 0.4 to 0.6 moderate, 0.6 to 0.8 strong, and tau-b values above 0.8 are considered very strong. Negative sign indicates inverse correlation. 95% confidence intervals were calculated. Significance was calculated with two-tailed tests, and throughout, p-values below 0.05 were considered significant. Independent t-tests were performed to analyze association of expression with clinicopathological variables and treatment response. For interpretation of the independent t test results’ clinical significance, effect size was assessed using Hedges’ gamma with 95% confidence intervals. All the significant results, suggesting a clinically applicable glycovariant analysis, were further tested with ROC analysis [22, 23] for which AUC values, 95% confidence intervals, and p values were calculated.

## Results

**Fig. 1:**
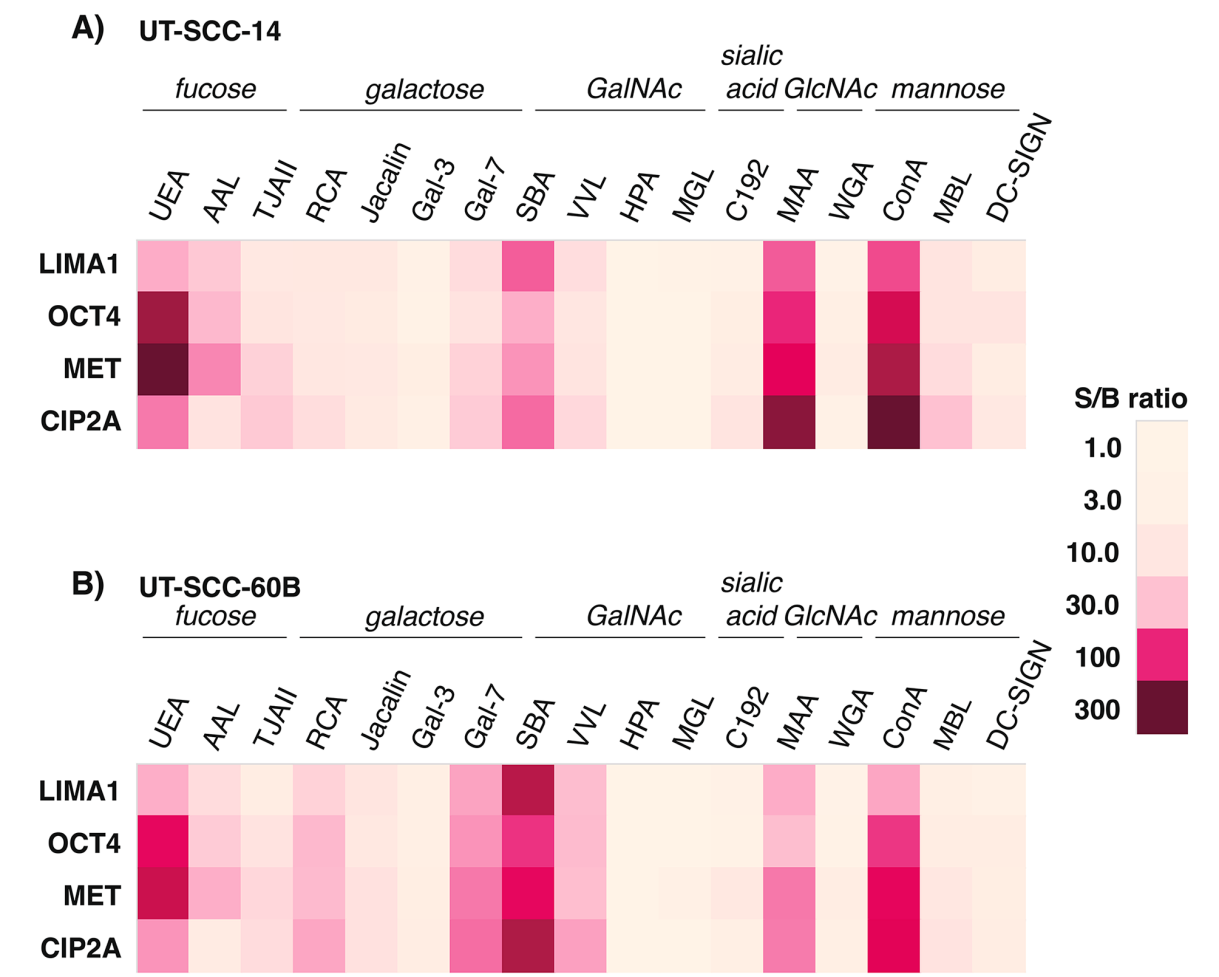
Signal-to-background (S/B) ratios from glycovariant screening of **(A)** UT-SCC-14, and **(B)** UT-SCC-60B cell lines using antibodies for stemness-related proteins LIMA1, OCT4, MET, and CIP2A. Lectin binding properties are summarized as italic headings, with SBA binding both galactose and GalNAc and MAA binding both sialic acid and GlcNAc.

The workflow of the study is depicted in Supplemental Fig. 1. First, a wide panel of lectins and other glycan-binding proteins (Table 1) targeting various sugar structures was used to screen the glycovariant expression of OCT4, MET, CIP2A, and LIMA1 using cell lysates of two patient-derived UT-SCC cell lines, UT-SCC-14 and UT-SCC-60B, and further their respective cell media. UT-SCC-14 is derived from grade II tongue squamous cell carcinoma, while UT-SCC-60B represents a metastatic squamous cell carcinoma of palatine tonsil. Both cell lines express OCT4 and CIP2A, both are p16 negative, and do not support high-risk HPV. The assay format resembles the conventional non-competitive, sandwich-type immunoassay, where the glycoprotein of interest is bound with biotinylated capture antibody on streptavidin plate and specific glycan structures detected with lectins and glycan-specific antibodies coated on Eu3+-NPs. Based on the signal-to-background (S/B) ratios in cell lysate screening, three of the most promising plant lectin-NPs (ConA, UEA, SBA), two mammalian lectin-NPs (DC-SIGN, MBL), and a glycan-specific Mab-NP (C192) were selected for further analysis of cell media. The results of the glycovariant screening of the two UT-SCC cell lines are presented in Fig. 1.

In both cell lines, protein glycovariants recognized by UEA, ConA, and SBA lectins were clearly the most abundant. Over two-fold difference in S/B ratios of the protein glycovariants between the two cell lines was observed with lectins MBL, SBA, VVL, RCA, Gal-7, and MAA. However, the S/B ratios with MBL and RCA were relatively small compared to other mannose- and galactose-specific lectins, namely ConA, UEA, and SBA. Thus, MBL and RCA were excluded from the following screening assays of tissue lysates. In both cell lines, HPA was negative with all four proteins and MGL was negative with three out of four proteins. On the other hand, the results of especially ConA and UEA, but also AAL assays were systematically increased across all four stemness-related proteins in cell lysates. The comparability of expression levels between cell lysates and cell media was confirmed using a condensed panel of lectins (Supplemental Table 1).

Based on the results of the cell line screening, a condensed panel of six lectins with the highest S/B ratios across both cell lines, and a good coverage of different glycan binding specificities, namely ConA, AAL, UEA, MAA, SBA, and VVL were chosen for the HNSCC tissue sample screening. Due to availability issues, VVL was substituted after careful analysis of substrate specificities with another GalNAc-specific lectin, WFL.

### Tumor versus normal

**Fig. 2:**
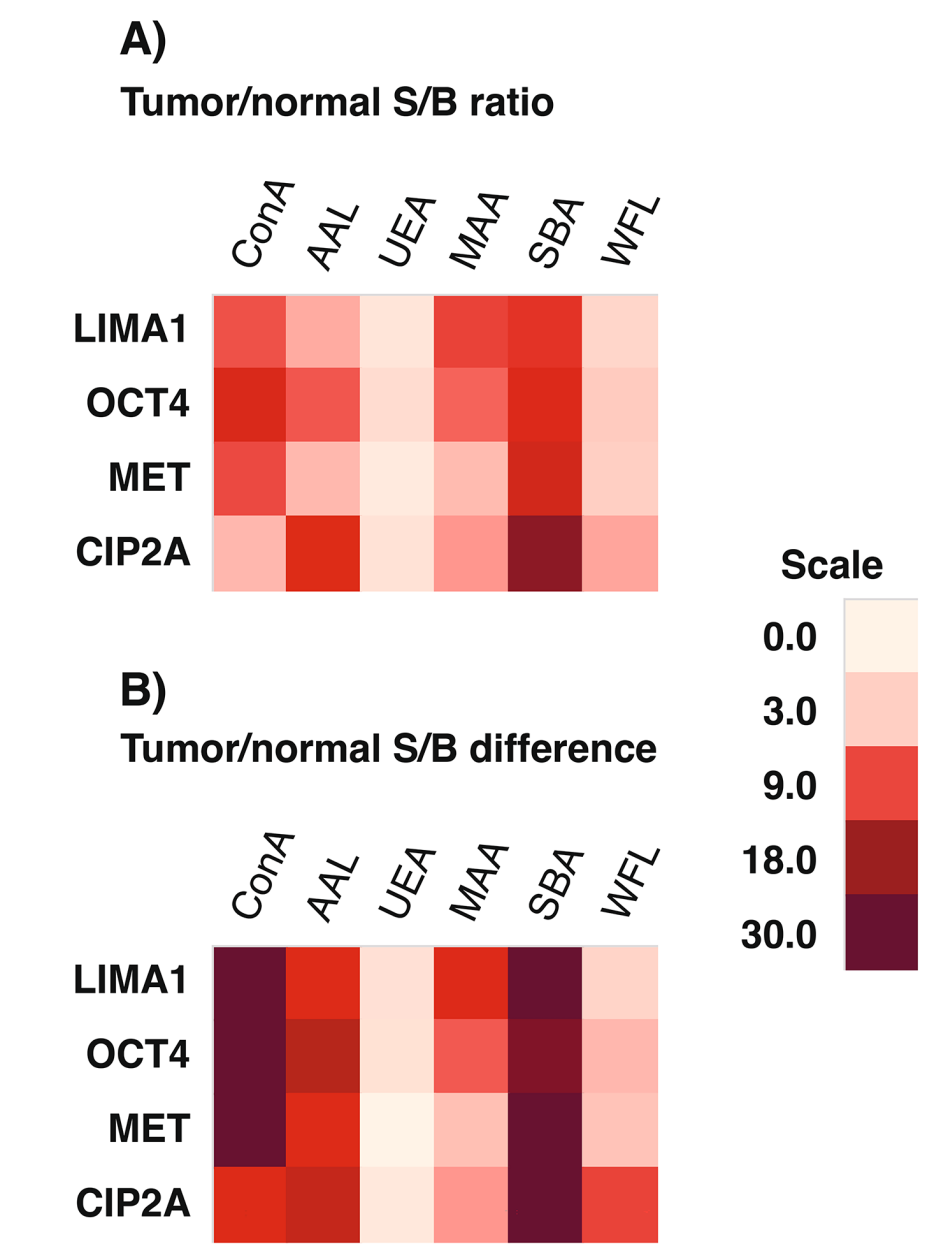
Tumor vs. normal signal-to-background (S/B) ratios from glycovariant screening of HNSCC tissue lysates (*N* = 25) **(A)** as a ratio of S/B ratios and **(B)** as a difference between S/B ratios

Next, to evaluate glycovariant expression in human HNSCC tissue samples, we collected tumor specimens from twenty-five patients with newly diagnosed HNSCC (Table 2). In addition, when surgically indicated, a specimen of adjacent normal tissue was collected. The tissue specimens were homogenized to release the proteins, and total protein concentration in BCA protein assay ranged between 239 and 848 µg/ml. In lectin bioaffinity assay, the S/B ratios of tumor tissue samples ranged from 1,0 to 224 (average 13,2, median 2,5). The most of the adjacent normal tissues showed S/B ratios close to background, with an average S/B 2,1 and median S/B 1,5. Using a paired t-test, the expression levels between tumor and normal tissue samples were assessed. Significantly increased tumor vs. normal tissue S/B ratios and S/B differences were observed in SBA and ConA assays across all four proteins, whereas notably, no significant increase was detected in UEA assays (Fig. 2).

**Table 2:** Characteristics of the patient cohort

	n	%
**Gender**		
*male*	14	56%
*female*	11	44%
**Age at diagnosis**		
*< 70*	13	52%
*> 70*	12	48%
**Smoker**		
*> 20 pack yrs*	17	68%
*< 20 pack yrs*	9	36%
**Alcohol consumption**		
*yes*	10	40%
*no*	16	64%
**Primary tumor site**		
*oral cavity*	17	68%
*oropharynx*	2	8%
*larynx*	5	20%
*hypopharynx*	1	4%
*other*	1	4%
**T class**		
*T0-2*	15	60%
*T3-4*	10	40%
**N class**		
*N0*	17	68%
*N+*	8	32%
**M class**		
*M0*	22	88%
*M+*	3	12%
**Recidive during follow-up**		
*yes*	6	24%
*no*	19	76%
**Treatment type**		
*Surgery only*	6	24%
*RT +/- surgery*	9	36%
*CRT +/- surgery*	10	40%

**Fig. 3:**
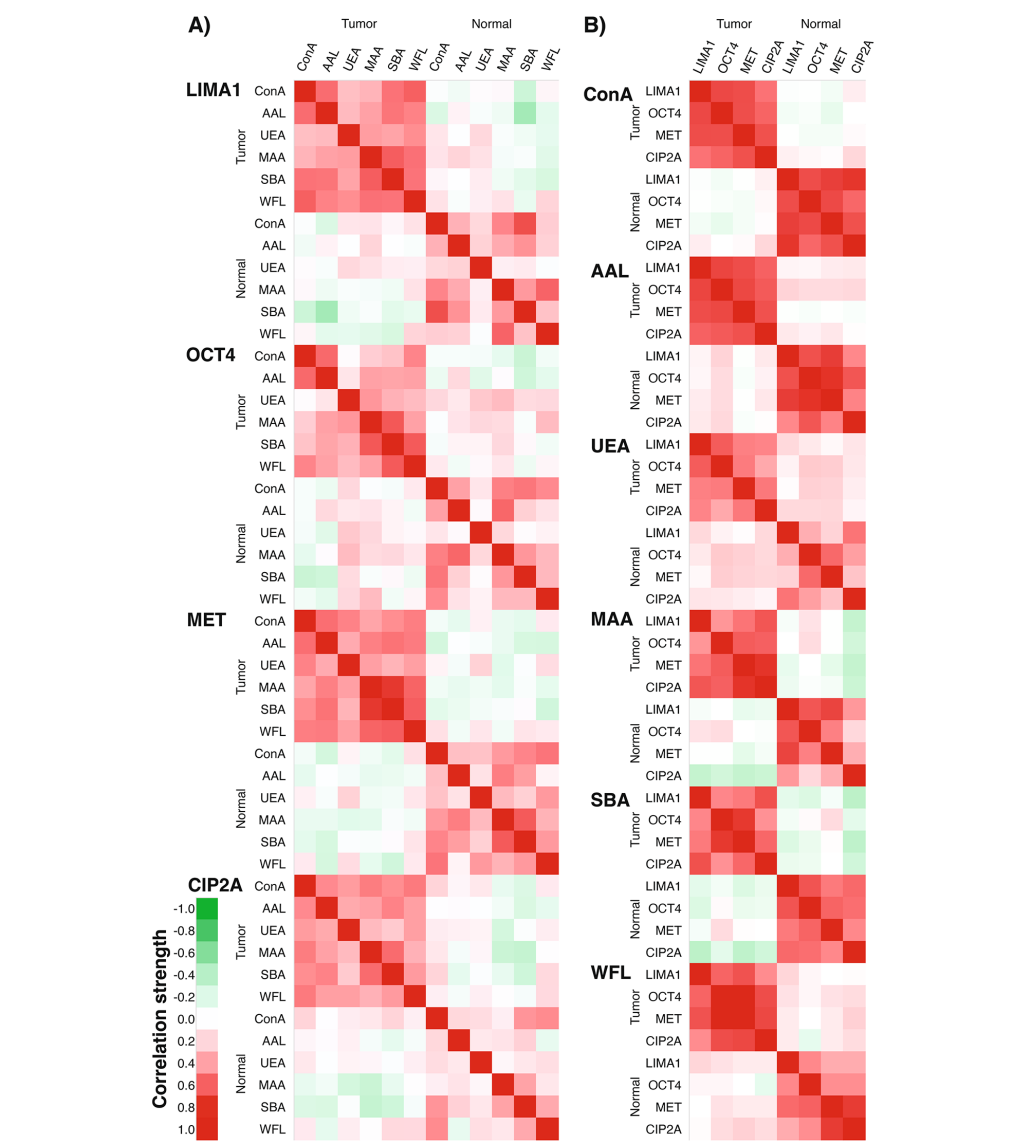
The correlations of tumor vs. tumor, tumor vs. normal and normal vs. normal samples from glycovariant screening of HNSCC tissue lysates (*N* = 25) **(A)** between six lectins and **(B)** between four stemness-related proteins

Next, the correlations between the six lectins and the four stemness-related proteins were assessed (Fig. 3, Supplemental Table 2). Very weak to weak correlations were observed between the glycovariant expression of tumor samples and normal samples, the only exception being LIMA1-AAL vs. LIMA1-SBA.

### Correlation between samples

Strong to very strong significant correlations with tau b values above 0.6 between different proteins with the same lectins were seen in tumor samples (Fig. 3, Supplemental Table 2). Especially strong correlation was observed between OCT4-WFL and MET-WFL, between MET-MAA and CIP2A-MAA and between OCT4-SBA and MET-SBA. Similar significant correlation between OCT4 and MET was seen with other lectins as well. Also in normal vs. normal sample groups, strong to very strong correlations were seen with ConA, AAL and SBA when comparing different proteins with the same lectins. Notable variation from weak to strong correlation was seen with especially UEA and MAA, but also with WFL. Importantly, very weak to weak correlation was observed between tumor and normal tissue samples with most lectin and protein combinations.

When comparing the same protein with different lectins mainly moderate correlations were noticed (Fig. 3A). Strong correlations were seen only with combinations MAA-SBA and SBA-WFL with LIMA1, OCT4 and MET, and the strongest correlation with MET-MAA and MET-SBA. In normal vs. normal comparison, the correlations varied from very weak to strong, with LIMA1-ConA and LIMA1-SBA and MET-MAA and MET-SBA demonstrating the strongest correlations.

### Predictive potential and association with clinicopathological characteristics

To analyze potential confounder bias, the dependence of the lectin assay results with key clinicopathological variables and comorbidities was assessed using effect size calculation of independent t-test (Supplemental Table 3). Patient age or sex did not correlate with any of the glycovariant assays. Importantly, there was no significant association between current alcohol use or alcohol exposure history and lectin assays, whereas current tobacco use was associated with several lectin assays, especially intratumoral CIP2A-UEA, -MAA, and -SBA, with significant Hedges γ of 0.89, 0.97, and 0.88, respectively. Tobacco history of over 20 pack-years, indicative of a canonical risk profile, was associated with intratumoral OCT4-WFL (γ=-0.75) and MET-SBA (γ=-0.62), reaching clinically borderline meaningful effect size. High T class was directly associated with intratumoral OCT4-WFL (γ=-1.05) and MET-WFL (γ=-1.03), whereas nodal positivity or the presence of distant metastasis was not significantly associated with any lectin assay. Interestingly, no significant association of the clinicopathological variables with lectin assay in adjacent macroscopically normal tissue was detected. No significant association was detected between the lectin assays and comorbidities.

To analyze the predictive potential of the lectin assay in treatment response, the association of lectin assays with radiotherapy response and tumor recurrence was investigated (Supplemental Table 3). Despite the association of stemness-related proteins with radiotherapy resistance, relatively weak associations between radiotherapy response and lectin assays were detected. Recurrence during follow-up was associated with especially SBA assays. However, using a ROC analysis, the power of the assays could not be verified (data not shown).

### Serum samples

The same condensed lectin panel was used to screen circulating glycovariants in HNSCC patient serum samples (Supplemental Table 4). Serum proved to be highly advantageous matrix since the background signals were very low ranging from 343 cps to 6798 cps (median 547 cps), with only AAL assays and CIP2A-UEA assays having background above 1500 cps. The S/B ratios varied between 2,2 and 234 (average 27,8, median 16,0), AAL resulting in highest S/B ratios. Mainly weak and non-significant correlations were observed between tumor tissue and serum sample groups. Between normal tissue and serum, strong correlations were seen only with OCT4 with five lectins, however, not reaching significance. Otherwise, weak to moderate correlations were observed between normal tissue and serum samples.

Similarly, as with tissue samples, the dependence of lectin assays with key clinicopathological variables and comorbidities were evaluated in serum samples. None of the assays were associated with patient age or sex. Interestingly, current alcohol or tobacco use, or alcohol or tobacco exposure history were not associated with any of the lectin assays. Cardiovascular disease was the only comorbidity associated with two lectin assays, namely LIMA1-MAA and MET-AAL. Nodal positivity was significantly associated with MET-UEA, MET-AAL, and LIMA1-AAL. Remarkably, distant metastasis was also significantly associated with MET-AAL. Serum LIMA1-AAL and MET-AAL indeed have a strong correlation (τ = 0.71; 95% CI: 0.45 to 0.86, *p* < 0.001), but neither correlates with the respective tumor expression (Supplemental Table 4).

## Discussion

This study describes the glycosylation changes of stemness-related putative biomarkers in HNSCC cell lines and patient samples. The correlation of changes in glycan expression across all four investigated proteins suggests a common glycosylation mechanism in these stemness-related proteins, which, importantly, is independent of the subcellular localization of the proteins. Cancer stem cells play an important role in several key mechanisms of cancer development, immune evasion, and therapy resistance [24]. The stemness of tumor cells can be assessed using multiple methods, such as expression of CD44, MET, OCT4 etc [5].. To our knowledge, this is the first study to evaluate stemness-related glycovariants in HNSCC. Importantly, we were able to identify a stemness-related mannose- and galactose-rich glycosylation niche in HNSCC, readily identified using ConA and SBA lectin-based assays. However, no definite association between radiotherapy response or cancer recurrence were detected, potentially due to the short follow-up period of this study.

In a previous study on HNSCC, fucosylation of PD-L2 was reported as a key finding, and its relation to therapy resistance was suggested [25]. Similarly, in oral cancer cell line OC2, a high rate of fucosylation has been reported [26]. Interestingly, while we demonstrate high expression of UEA-recognized fucosylated glycovariants of the stemness markers in cell lines, very similar expression between tumor and normal tissue was observed, suggesting that fucosylation of the stemness markers may not be restricted to the tumor and may rather be indicative of field carcinogenesis and exposure effects. Similarly, an increase in total fucose levels in serum of HNSCC patients has been reported [27, 28], and indeed in our study as well, fucosylated glycovariants recognized by AAL lectin were the most abundant glycovariants in serum.

Another study using a lectin-based electrophoresis method demonstrated overexpression of mannose-glycosylated AAT and APOA1 were significantly upregulated in serum of oral squamous cell carcinoma (OSCC) patients [29]. Importantly, a recent analysis reported oligomannose-glycans to be the most common N-glycan structures, while sialylated complex-type glycans and fucosylated glycans were less common alterations in HNSCC cell lines [30]. This supports our findings that glycan structures recognized by ConA lectin, known to have a strong binding affinity to oligomannose- and hybrid-type mannose-containing N-glycans, were most abundant in HNSCC cell lines as well as in HNSCC tissue samples across all four stemness-related proteins. Similar findings have been reported in various cancers [31, 32].

In several of our previous investigations, we have demonstrated that nanoparticle-aided lectin bioaffinity assays could be used in serum screening in the context of detection of cancers such as ovarian, prostate and colorectal cancer [17, 33, 34]. In this study, the association of stemness-related biomarker glycovariants in HNSCC tumor tissue, normal adjacent tissue and serum was evaluated. We found no significant correlation in stemness-related biomarker glycovariant levels between tumor tissue and serum or tumor tissue and adjacent normal tissue, but weak to moderate correlation was observed between normal tissue and serum. Only OCT4 glycovariants had strong albeit non-significant correlation between normal tissue and serum. MET-UEA, MET-AAL and LIMA1-AAL in serum were found to be associated with nodal positivity and MET-AAL also with distant metastasis. However, MET-AAL was associated also with patient cardiovascular disease, a major confounder that might introduce bias. Here, we have shown that although stemness-related protein biomarkers are found in blood circulation in higher-than-expected concentrations, their glycovariant levels in blood do not seem to be associated with cancer behavior. Therefore, we hypothesize that the leakage of stemness-related protein biomarkers from tumor tissue to adjacent tissue and further to blood circulation is restricted. Thus, based on our study, we conclude that it is unlikely, that further serum-based glycovariant screening of stemness-related biomarkers would yield significant applicability in the context of detection of HNSCC or therapy stratification of diagnosed cases.

The representativeness of cell lines in comparison with the actual tumors is a hot topic [35, 36]. In this study, we show that our main finding, the ConA-identifiable mannose- and SBA-identifiable galactose-glycosylations of stemness-related proteins, especially OCT4 and MET, is readily seen in both investigated patient-derived UT-SCC cell lines. In fact, this is well in line with our previous finding of a relationship between OCT4-related intrinsic radioresistance in a wide panel of UT-SCC cell lines and clinical radioresistance in a carefully constructed patient material [5, 8]. However, we emphasize the need for repeating screens performed in cell lines in a representative patient material. Recently, LIMA1, previously described as a cytoskeleton linked membrane protein and prognostic marker in several cancer types, has been linked to pluripotency regulation [37]. Importantly, the observed similarity of the glycovariant profile of LIMA1 with the well-accepted stemness proteins provides evidence of a stemness-related role for LIMA1 in HNSCC, associated with a stemness-related glycosylation niche rich in mannose and galactose.

Glycan assays based on lectins have been widely used to discover and detect new disease-related glycosylation changes [38]. The introduction of assays utilizing the combined protein-glycan specificity of antibodies and lectins, such as enzyme-linked lectin assays [39], has led to a myriad of analytical applications for the detection of various diseases. While powerful tools in glycovariant discovery, a key limitation of lectins is their intrinsically broad specificity to the respective glycan target, which can lead to unwanted cross-reactivity with other glycans and reduce the sensitivity and accuracy of the assay. Notwithstanding, lectin-based assays remain important for studying glycosylation patterns in the context of biomarker discovery and proof-of-concept assay development. In studies with processed fresh tissue samples, the variation in homogenization process may impact the reproducibility of individual lectin bioaffinity assay results. However, it is noteworthy that in this study, there were almost 300-fold (S/B ratios ranging between 1,0 and 296) differences in expression levels of different glycovariants, making it unlikely that the observed effects would significantly be caused by preanalytical errors.

## Conclusions

In line with previous studies, we report mannose- and galactose-rich glycosylation in stemness-related proteins implicated in radioresistance development in HNSCC tumor tissue. However, due to confounding factors, we conclude that such alterations rarely correlate with serum-level expression, emphasizing the need for high-quality tissue sample studies.

### Electronic supplementary material

Below is the link to the electronic supplementary material.

Supplementary Material 1

Supplementary Material 2

Supplementary Material 3

Supplementary Material 4

Supplementary Material 5

## Data Availability

Data is available from the corresponding author upon a reasonable request.
