# Supplementary figures and images for: Identification of stemness-related glycosylation changes in head and neck squamous cell carcinoma
Source: BMC Cancer. 2024 Apr 10;24:443. doi: 10.1186/s12885-024-12161-5 (PMC11005150; doi:10.1186/s12885-024-12161-5)

**Glycovariant screening of stemness-related proteins**  
*OCT4, CIP2A, MET, LIMA1*

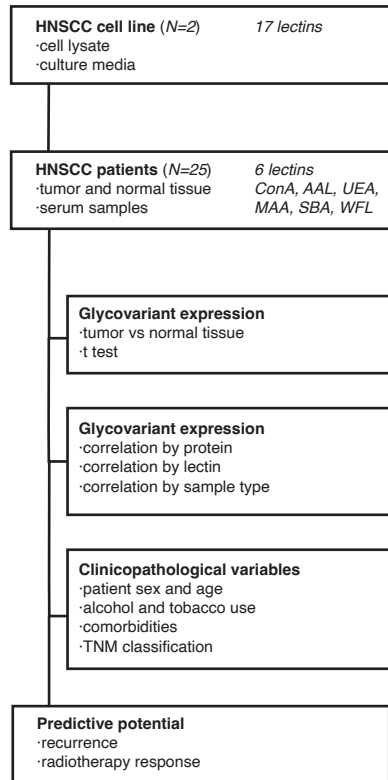

Supplement: Supplementary file 1 — Supplementary Material 1 [file 12885_2024_12161_MOESM1_ESM.pdf]
