# Supplementary material for: Identification of stemness-related glycosylation changes in head and neck squamous cell carcinoma
Source: BMC Cancer. 2024 Apr 10;24:443. doi: 10.1186/s12885-024-12161-5 (PMC11005150; doi:10.1186/s12885-024-12161-5)
Supplement: Supplementary file 2 — Supplementary Material 2 [file 12885_2024_12161_MOESM2_ESM.pdf]

Supplemental table 1:

### *Cell lysates - total lectin panel - sg/bg ratios:*

Cell lysates (1:500 dilution):

| | UEA | | DC-SIGN | | MGL | | MBL | | C192 | | WGA | |
|---|---|---|---|---|---|---|---|---|---|---|---|---|
| | 14 | 60B | 14 | 60B | 14 | 60B | 14 | 60B | 14 | 60B | 14 | 60B |
| **LIMA** | 39,4 | 38,4 | 4,7 | 2,9 | 2,0 | 1,4 | 10,9 | 3,6 | 3,0 | 2,5 | 1,7 | 1,5 |
| **OCT4** | 198,5 | 124,0 | 10,9 | 4,6 | 0,9 | 0,8 | 10,9 | 4,7 | 4,1 | 2,1 | 3,9 | 2,1 |
| **MET** | 296,2 | 163,2 | 5,5 | 4,1 | 1,2 | 3,3 | 15,9 | 9,3 | 5,9 | 8,3 | 5,9 | 4,7 |
| **CIP2A** | 60,8 | 49,9 | 9,0 | 5,4 | 1,3 | 1,1 | 29,7 | 11,1 | 10,6 | 3,9 | 2,9 | 3,7 |

| | TJA II | | AAL | | CON A | | Gal_3 | | SBA | | HPA | |
|---|---|---|---|---|---|---|---|---|---|---|---|---|
| | 14 | 60B | 14 | 60B | 14 | 60B | 14 | 60B | 14 | 60B | 14 | 60B |
| **LIMA** | 9,0 | 5,5 | 27,0 | 15,7 | 83,5 | 42,4 | 2,6 | 3,7 | 74,1 | 179,1 | 1,1 | 1,0 |
| **OCT4** | 10,2 | 11,7 | 33,7 | 25,1 | 154,4 | 91,9 | 2,3 | 3,6 | 39,1 | 93,3 | 1,1 | 1,0 |
| **MET** | 21,7 | 18,7 | 56,4 | 39,1 | 188,2 | 126,2 | 4,1 | 4,4 | 50,3 | 124,2 | 1,1 | 1,1 |
| **CIP2A** | 26,3 | 16,3 | 11,6 | 7,0 | 259,9 | 137,3 | 3,0 | 4,7 | 68,0 | 185,8 | 1,0 | 1,1 |

| | Jacalin | | VVL | | RCA | | Gal-7 | | MAA | |
|---|---|---|---|---|---|---|---|---|---|---|
| | 14 | 60B | 14 | 60B | 14 | 60B | 14 | 60B | 14 | 60B |
| **LIMA** | 8,3 | 10,2 | 14,7 | 31,7 | 8,0 | 21,1 | 15,7 | 43,6 | 75,2 | 39,7 |
| **OCT4** | 6,4 | 8,1 | 10,0 | 32,6 | 8,8 | 33,8 | 11,7 | 50,6 | 98,9 | 31,1 |
| **MET** | 8,9 | 12,9 | 10,9 | 31,6 | 9,4 | 33,0 | 21,5 | 61,4 | 129,7 | 61,4 |
| **CIP2A** | 7,3 | 12,9 | 18,5 | 45,1 | 15,4 | 42,4 | 25,1 | 67,2 | 216,7 | 60,2 |

| |
|---|
| 1,00 |
| 3,00 |
| 10,00 |
| 30,0 |
| 100,00 |
| 300,00 |

### *Cell lysates vs. cell culture media - condenced lectin panel - sg/bg ratios:*

Cell lysates (1:500 dilution):

| | UEA | | DC-SIGN | | MBL | | C192 | | CON A | | SBA | |
|---|---|---|---|---|---|---|---|---|---|---|---|---|
| | 14 | 60B | 14 | 60B | 14 | 60B | 14 | 60B | 14 | 60B | 14 | 60B |
| **LIMA** | 39,4 | 38,4 | 4,7 | 2,9 | 10,9 | 3,6 | 3,0 | 2,5 | 83,5 | 42,4 | 74,1 | 179,1 |
| **OCT4** | 198,5 | 124,0 | 10,9 | 4,6 | 10,9 | 4,7 | 4,1 | 2,1 | 154,4 | 91,9 | 39,1 | 93,3 |
| **MET** | 296,2 | 163,2 | 5,5 | 4,1 | 15,9 | 9,3 | 5,9 | 8,3 | 188,2 | 126,2 | 50,3 | 124,2 |
| **CIP2A** | 60,8 | 49,9 | 9,0 | 5,4 | 29,7 | 11,1 | 10,6 | 3,9 | 259,9 | 137,3 | 68,0 | 185,8 |

| |
|---|
| 1,00 |
| 3,00 |
| 10,00 |
| 30,0 |
| 100,00 |
| 300,00 |

Cell culture media (1:2 dilution):

| | UEA | | DC-SIGN | | MBL | | C192 | | CON A | | SBA | |
|---|---|---|---|---|---|---|---|---|---|---|---|---|
| | 14 | 60B | 14 | 60B | 14 | 60B | 14 | 60B | 14 | 60B | 14 | 60B |
| **LIMA** | 8,9 | 8,3 | 0,9 | 0,9 | 1,0 | 0,9 | 1,3 | 1,6 | 11,7 | 5,4 | 15,8 | 7,9 |
| **OCT4** | 19,5 | 18,7 | 1,2 | 1,0 | 1,0 | 1,0 | 2,2 | 1,0 | 20,4 | 10,5 | 47,2 | 12,1 |
| **MET** | 14,5 | 11,0 | 0,8 | 1,2 | 1,1 | 1,0 | 1,6 | 1,0 | 13,1 | 7,4 | 33,2 | 9,0 |
| **CIP2A** | 11,2 | 7,8 | 0,6 | 0,8 | 1,1 | 0,9 | 1,6 | 0,9 | 16,0 | 8,5 | 24,2 | 9,5 |
