# Supplementary material for: Identification of stemness-related glycosylation changes in head and neck squamous cell carcinoma
Source: BMC Cancer. 2024 Apr 10;24:443. doi: 10.1186/s12885-024-12161-5 (PMC11005150; doi:10.1186/s12885-024-12161-5)
Supplement: Supplementary file 3 — Supplementary Material 3 [file 12885_2024_12161_MOESM3_ESM.pdf]

Supplemental Table 2. **Correlation between expression levels of the investigated protein-lectin pairs in tumor tissue lysates.**

BY PROTEIN:

	Protein-lectin pairs	correlation tau b	95% CI lower limit	upper limit	P
**LIMA1:**	LIMA1_ConA - LIMA1_AAL	.560	.336	.724	<.001
	LIMA1_ConA - LIMA1_UEA	.297	.017	.534	.042
	LIMA1_ConA - LIMA1_MAA	.333	.041	.573	.030
	LIMA1_ConA - LIMA1_SBA	.541	.292	.722	<.001
	LIMA1_ConA - LIMA1_WFL	.602	.347	.774	<.001
	LIMA1_ConA - N_LIMA1_ConA	-.020	-.350	.316	.910
	LIMA1_ConA - N_LIMA1_AAL	-.085	-.407	.255	.622
	LIMA1_ConA - N_LIMA1_UEA	.020	-.316	.350	.910
	LIMA1_ConA - N_LIMA1_MAA	.033	-.304	.362	.850
	LIMA1_ConA - N_LIMA1_SBA	-.294	-.580	.056	.099
	LIMA1_ConA - N_LIMA1_WFL	.050	-.313	.400	.787
	LIMA1_AAL - LIMA1_UEA	.313	.041	.542	.028
	LIMA1_AAL - LIMA1_MAA	.399	.125	.617	.008
	LIMA1_AAL - LIMA1_SBA	.534	.289	.713	<.001
	LIMA1_AAL - LIMA1_WFL	.495	.215	.699	.002
	LIMA1_AAL - N_LIMA1_ConA	-.216	-.504	.114	.196
	LIMA1_AAL - N_LIMA1_AAL	.064	-.264	.379	.700
	LIMA1_AAL - N_LIMA1_UEA	-.041	-.359	.285	.807
	LIMA1_AAL - N_LIMA1_MAA	-.099	-.409	.231	.552
	LIMA1_AAL - N_LIMA1_SBA	-.477	-.699	-.171	.006
	LIMA1_AAL - N_LIMA1_WFL	-.162	-.480	.194	.365
	LIMA1_UEA - LIMA1_MAA	.415	.143	.628	.006
	LIMA1_UEA - LIMA1_SBA	.391	.116	.611	.009
	LIMA1_UEA - LIMA1_WFL	.463	.176	.678	.004
	LIMA1_UEA - N_LIMA1_ConA	.124	-.218	.439	.472
	LIMA1_UEA - N_LIMA1_AAL	-.007	-.339	.327	.970
	LIMA1_UEA - N_LIMA1_UEA	.190	-.153	.492	.272
	LIMA1_UEA - N_LIMA1_MAA	-.033	-.362	.304	.850
	LIMA1_UEA - N_LIMA1_SBA	-.088	-.420	.264	.621
	LIMA1_UEA - N_LIMA1_WFL	-.150	-.482	.219	.418
	LIMA1_MAA - LIMA1_SBA	.610	.383	.768	<.001
	LIMA1_MAA - LIMA1_WFL	.544	.268	.737	.001
	LIMA1_MAA - N_LIMA1_ConA	.132	-.222	.456	.458
	LIMA1_MAA - N_LIMA1_AAL	.206	-.149	.514	.249
	LIMA1_MAA - N_LIMA1_UEA	.147	-.208	.468	.410
	LIMA1_MAA - N_LIMA1_MAA	-.059	-.395	.292	.742
	LIMA1_MAA - N_LIMA1_SBA	-.033	-.386	.328	.857
	LIMA1_MAA - N_LIMA1_WFL	-.181	-.518	.205	.347
	LIMA1_SBA - LIMA1_WFL	.537	.269	.728	<.001
	LIMA1_SBA - N_LIMA1_ConA	.044	-.305	.383	.805
	LIMA1_SBA - N_LIMA1_AAL	.000	-.345	.345	1.000
	LIMA1_SBA - N_LIMA1_UEA	.088	-.264	.420	.621
	LIMA1_SBA - N_LIMA1_MAA	-.088	-.420	.264	.621
	LIMA1_SBA - N_LIMA1_SBA	-.167	-.495	.203	.368
	LIMA1_SBA - N_LIMA1_WFL	-.238	-.560	.147	.216
	LIMA1_WFL - N_LIMA1_ConA	.143	-.260	.503	.477
	LIMA1_WFL - N_LIMA1_AAL	-.033	-.416	.360	.870
	LIMA1_WFL - N_LIMA1_UEA	.077	-.321	.452	.702
	LIMA1_WFL - N_LIMA1_MAA	.143	-.260	.503	.477
	LIMA1_WFL - N_LIMA1_SBA	-.128	-.509	.294	.542
	LIMA1_WFL - N_LIMA1_WFL	.205	-.220	.565	.329
	N_LIMA1_ConA - N_LIMA1_AAL	.345	.025	.601	.039
	N_LIMA1_ConA - N_LIMA1_UEA	.193	-.138	.485	.248
	N_LIMA1_ConA - N_LIMA1_MAA	.485	.193	.699	.004
	N_LIMA1_ConA - N_LIMA1_SBA	.660	.419	.814	<.001
	N_LIMA1_ConA - N_LIMA1_WFL	.235	-.119	.536	.187
	N_LIMA1_AAL - N_LIMA1_UEA	.216	-.114	.504	.196
	N_LIMA1_AAL - N_LIMA1_MAA	.368	.052	.618	.028
	N_LIMA1_AAL - N_LIMA1_SBA	.451	.139	.682	.009
	N_LIMA1_AAL - N_LIMA1_WFL	.221	-.134	.525	.217
	N_LIMA1_UEA - N_LIMA1_MAA	.076	-.253	.389	.649
	N_LIMA1_UEA - N_LIMA1_SBA	.098	-.243	.417	.570
	N_LIMA1_UEA - N_LIMA1_WFL	-.015	-.358	.332	.934
	N_LIMA1_MAA - N_LIMA1_SBA	.425	.107	.664	.014
	N_LIMA1_MAA - N_LIMA1_WFL	.588	.306	.776	<.001
	N_LIMA1_SBA - N_LIMA1_WFL	.279	-.072	.569	.118
**OCT4:**	OCT4_ConA - OCT4_AAL	.573	.354	.733	<.001
	OCT4_ConA - OCT4_UEA	.020	-.271	.307	.895
	OCT4_ConA - OCT4_MAA	.238	-.063	.499	.121
	OCT4_ConA - OCT4_SBA	.276	-.031	.535	.080
	OCT4_ConA - OCT4_WFL	.485	.169	.711	.007
	OCT4_ConA - N_OCT4_ConA	-.046	-.373	.292	.791
	OCT4_ConA - N_OCT4_AAL	-.046	-.373	.292	.791
	OCT4_ConA - N_OCT4_UEA	-.059	-.384	.280	.733
	OCT4_ConA - N_OCT4_MAA	-.150	-.460	.192	.384
	OCT4_ConA - N_OCT4_SBA	-.309	-.591	.040	.084
	OCT4_ConA - N_OCT4_WFL	-.077	-.452	.321	.702
	OCT4_AAL - OCT4_UEA	.130	-.157	.398	.372
	OCT4_AAL - OCT4_MAA	.399	.125	.617	.008
	OCT4_AAL - OCT4_SBA	.385	.101	.612	.012
	OCT4_AAL - OCT4_WFL	.399	.076	.646	.021
	OCT4_AAL - N_OCT4_ConA	-.111	-.428	.230	.520
	OCT4_AAL - N_OCT4_AAL	.181	-.150	.476	.278
	OCT4_AAL - N_OCT4_UEA	-.181	-.476	.150	.278
	OCT4_AAL - N_OCT4_MAA	.018	-.307	.338	.916
	OCT4_AAL - N_OCT4_SBA	-.281	-.562	.057	.103
	OCT4_AAL - N_OCT4_WFL	-.162	-.504	.223	.400
	OCT4_UEA - OCT4_MAA	.439	.172	.646	.003
	OCT4_UEA - OCT4_SBA	.342	.051	.580	.026
	OCT4_UEA - OCT4_WFL	.307	-.029	.581	.075
	OCT4_UEA - N_OCT4_ConA	.191	-.164	.503	.284
	OCT4_UEA - N_OCT4_AAL	.111	-.230	.428	.520
	OCT4_UEA - N_OCT4_UEA	.255	-.085	.542	.140
	OCT4_UEA - N_OCT4_MAA	.307	-.029	.581	.075
	OCT4_UEA - N_OCT4_SBA	.176	-.179	.491	.323
	OCT4_UEA - N_OCT4_WFL	.187	-.217	.536	.352
	OCT4_MAA - OCT4_SBA	.628	.407	.779	<.001
	OCT4_MAA - OCT4_WFL	.399	.076	.646	.021
	OCT4_MAA - N_OCT4_ConA	-.017	-.372	.343	.928
	OCT4_MAA - N_OCT4_AAL	.132	-.222	.456	.458
	OCT4_MAA - N_OCT4_UEA	.191	-.164	.503	.284
	OCT4_MAA - N_OCT4_MAA	.176	-.179	.491	.323
	OCT4_MAA - N_OCT4_SBA	-.033	-.386	.328	.857
	OCT4_MAA - N_OCT4_WFL	.308	-.113	.635	.143
	OCT4_SBA - OCT4_WFL	.621	.363	.791	<.001
	OCT4_SBA - N_OCT4_ConA	-.050	-.400	.313	.787
	OCT4_SBA - N_OCT4_AAL	.059	-.292	.395	.742
	OCT4_SBA - N_OCT4_UEA	.059	-.292	.395	.742
	OCT4_SBA - N_OCT4_MAA	.191	-.164	.503	.284
	OCT4_SBA - N_OCT4_SBA	.033	-.328	.386	.857
	OCT4_SBA - N_OCT4_WFL	-.077	-.469	.341	.714

BY LECTIN:

	Protein-lectin pairs	correlation tau b	95% CI lower limit	upper limit	P
**ConA:**	LIMA1_ConA - OCT4_ConA	.687	.507	.809	<.001
	LIMA1_ConA - MET_ConA	.673	.488	.800	<.001
	LIMA1_ConA - CIP2A_ConA	.547	.319	.715	<.001
	OCT4_ConA - MET_ConA	.667	.479	.796	<.001
	OCT4_ConA - CIP2A_ConA	.593	.380	.747	<.001
	MET_ConA - CIP2A_ConA	.660	.470	.792	<.001
	LIMA1_ConA - N_OCT4_ConA	-.007	-.339	.327	.970
	LIMA1_ConA - N_MET_ConA	-.085	-.407	.255	.622
	LIMA1_ConA - N_CIP2A_ConA	.098	-.243	.417	.570
	OCT4_ConA - N_LIMA1_ConA	-.085	-.407	.255	.622
	OCT4_ConA - N_MET_ConA	-.150	-.460	.192	.384
	OCT4_ConA - N_CIP2A_ConA	.007	-.327	.339	.970
	MET_ConA - N_LIMA1_ConA	-.007	-.339	.327	.970
	MET_ConA - N_OCT4_ConA	-.072	-.395	.268	.677
	MET_ConA - N_CIP2A_ConA	.033	-.304	.362	.850
	CIP2A_ConA - N_LIMA1_ConA	.085	-.255	.407	.622
	CIP2A_ConA - N_OCT4_ConA	.046	-.292	.373	.791
	CIP2A_ConA - N_MET_ConA	.020	-.316	.350	.910
	N_LIMA1_ConA - N_OCT4_ConA	.673	.438	.822	<.001
	N_LIMA1_ConA - N_MET_ConA	.725	.517	.853	<.001
	N_LIMA1_ConA - N_CIP2A_ConA	.754	.571	.866	<.001
	N_OCT4_ConA - N_MET_ConA	.686	.458	.830	<.001
	N_OCT4_ConA - N_CIP2A_ConA	.582	.308	.766	<.001
	N_MET_ConA - N_CIP2A_ConA	.660	.419	.814	<.001
**AAL:**	LIMA1_AAL - OCT4_AAL	.705	.537	.819	<.001
	LIMA1_AAL - MET_AAL	.674	.494	.798	<.001
	LIMA1_AAL - CIP2A_AAL	.600	.394	.748	<.001
	OCT4_AAL - MET_AAL	.698	.528	.815	<.001
	OCT4_AAL - CIP2A_AAL	.625	.427	.765	<.001
	MET_AAL - CIP2A_AAL	.655	.469	.786	<.001
	LIMA1_AAL - N_OCT4_AAL	.053	-.275	.369	.753
	LIMA1_AAL - N_MET_AAL	.099	-.231	.409	.552
	LIMA1_AAL - N_CIP2A_AAL	.111	-.219	.419	.506
	OCT4_AAL - N_LIMA1_AAL	.216	-.114	.504	.196
	OCT4_AAL - N_MET_AAL	.181	-.150	.476	.278
	OCT4_AAL - N_CIP2A_AAL	.193	-.138	.485	.248
	MET_AAL - N_LIMA1_AAL	-.018	-.338	.307	.916
	MET_AAL - N_OCT4_AAL	-.053	-.369	.275	.753
	MET_AAL - N_CIP2A_AAL	-.041	-.359	.285	.807
	CIP2A_AAL - N_LIMA1_AAL	.111	-.219	.419	.506
	CIP2A_AAL - N_OCT4_AAL	.076	-.253	.389	.649
	CIP2A_AAL - N_MET_AAL	.123	-.208	.428	.463
	N_LIMA1_AAL - N_OCT4_AAL	.661	.430	.811	<.001
	N_LIMA1_AAL - N_MET_AAL	.731	.534	.853	<.001
	N_LIMA1_AAL - N_CIP2A_AAL	.485	.193	.699	.004
	N_OCT4_AAL - N_MET_AAL	.766	.589	.873	<.001
	N_OCT4_AAL - N_CIP2A_AAL	.637	.396	.796	<.001
	N_MET_AAL - N_CIP2A_AAL	.497	.208	.706	.003
**UEA:**	LIMA1_UEA - OCT4_UEA	.623	.414	.770	<.001
	LIMA1_UEA - MET_UEA	.507	.269	.686	<.001
	LIMA1_UEA - CIP2A_UEA	.493	.252	.677	<.001
	OCT4_UEA - MET_UEA	.522	.281	.701	<.001
	OCT4_UEA - CIP2A_UEA	.377	.106	.595	.010
	MET_UEA - CIP2A_UEA	.533	.302	.705	<.001
	LIMA1_UEA - N_OCT4_UEA	.111	-.230	.428	.520
	LIMA1_UEA - N_MET_UEA	.046	-.292	.373	.791
	LIMA1_UEA - N_CIP2A_UEA	.137	-.205	.450	.426
	OCT4_UEA - N_LIMA1_UEA	.072	-.268	.395	.677
	OCT4_UEA - N_MET_UEA	.242	-.099	.532	.161
	OCT4_UEA - N_CIP2A_UEA	.124	-.218	.439	.472
	MET_UEA - N_LIMA1_UEA	.020	-.316	.350	.910
	MET_UEA - N_OCT4_UEA	.229	-.113	.522	.185
	MET_UEA - N_CIP2A_UEA	.098	-.243	.417	.570
	CIP2A_UEA - N_LIMA1_UEA	.190	-.153	.492	.272
	CIP2A_UEA - N_OCT4_UEA	.190	-.153	.492	.272
	CIP2A_UEA - N_MET_UEA	.203	-.140	.502	.240
	N_LIMA1_UEA - N_OCT4_UEA	.357	.039	.609	.033
	N_LIMA1_UEA - N_MET_UEA	.216	-.114	.504	.196
	N_LIMA1_UEA - N_CIP2A_UEA	.544	.268	.737	.001
	N_OCT4_UEA - N_MET_UEA	.579	.315	.760	<.001
	N_OCT4_UEA - N_CIP2A_UEA	.415	.107	.651	.013
	N_MET_UEA - N_CIP2A_UEA	.275	-.052	.549	.100
**MAA:**	LIMA1_MAA - OCT4_MAA	.447	.181	.651	.003
	LIMA1_MAA - MET_MAA	.542	.300	.718	<.001
	LIMA1_MAA - CIP2A_MAA	.636	.426	.782	<.001
	OCT4_MAA - MET_MAA	.621	.404	.771	<.001
	OCT4_MAA - CIP2A_MAA	.605	.383	.761	<.001
	MET_MAA - CIP2A_MAA	.763	.608	.862	<.001
	LIMA1_MAA - N_OCT4_MAA	.147	-.208	.468	.410
	LIMA1_MAA - N_MET_MAA	-.015	-.358	.332	.934
	LIMA1_MAA - N_CIP2A_MAA	-.338	-.612	.007	.058
	OCT4_MAA - N_LIMA1_MAA	.000	-.345	.345	1.000
	OCT4_MAA - N_MET_MAA	-.015	-.358	.332	.934
	OCT4_MAA - N_CIP2A_MAA	-.279	-.569	.072	.118
	MET_MAA - N_LIMA1_MAA	-.147	-.468	.208	.410
	MET_MAA - N_OCT4_MAA	.000	-.345	.345	1.000
	MET_MAA - N_CIP2A_MAA	-.338	-.612	.007	.058
	CIP2A_MAA - N_LIMA1_MAA	-.118	-.444	.237	.510
	CIP2A_MAA - N_OCT4_MAA	-.029	-.370	.318	.869
	CIP2A_MAA - N_MET_MAA	-.074	-.408	.278	.680
	N_LIMA1_MAA - N_OCT4_MAA	.637	.396	.796	<.001
	N_LIMA1_MAA - N_MET_MAA	.708	.499	.839	<.001
	N_LIMA1_MAA - N_CIP2A_MAA	.439	.135	.667	.009
	N_OCT4_MAA - N_MET_MAA	.509	.223	.714	.002
	N_OCT4_MAA - N_CIP2A_MAA	.193	-.138	.485	.248
	N_MET_MAA - N_CIP2A_MAA	.357	.039	.609	.033
**SBA:**	LIMA1_SBA - OCT4_SBA	.489	.226	.686	.001
	LIMA1_SBA - MET_SBA	.526	.279	.707	<.001
	LIMA1_SBA - CIP2A_SBA	.660	.459	.797	<.001
	OCT4_SBA - MET_SBA	.749	.582	.855	<.001
	OCT4_SBA - CIP2A_SBA	.455	.183	.662	.003
	MET_SBA - CIP2A_SBA	.581	.351	.745	<.001
	LIMA1_SBA - N_OCT4_SBA	-.217	-.533	.153	.242
	LIMA1_SBA - N_MET_SBA	-.067	-.414	.298	.719
	LIMA1_SBA - N_CIP2A_SBA	-.383	-.652	-.030	.038
	OCT4_SBA - N_LIMA1_SBA	-.083	-.428	.283	.653
	OCT4_SBA - N_MET_SBA	.183	-.186	.508	.322
	OCT4_SBA - N_CIP2A_SBA	-.167	-.495	.203	.368
	MET_SBA - N_LIMA1_SBA	-.217	-.533	.153	.242
	MET_SBA - N_OCT4_SBA	-.133	-.469	.235	.471
	MET_SBA - N_CIP2A_SBA	-.367	-.640	-.011	.048

	OCT4_WFL - N_OCT4_ConA	.103	-.318	.489	.625
	OCT4_WFL - N_OCT4_AAL	-.077	-.452	.321	.702
	OCT4_WFL - N_OCT4_UEA	.033	-.360	.416	.870
	OCT4_WFL - N_OCT4_MAA	.121	-.281	.486	.547
	OCT4_WFL - N_OCT4_SBA	-.103	-.489	.318	.625
	OCT4_WFL - N_OCT4_WFL	.051	-.363	.449	.807
	N_OCT4_ConA - N_OCT4_AAL	.399	.076	.646	.021
	N_OCT4_ConA - N_OCT4_UEA	.124	-.218	.439	.472
	N_OCT4_ConA - N_OCT4_MAA	.503	.204	.716	.004
	N_OCT4_ConA - N_OCT4_SBA	.529	.226	.739	.003
	N_OCT4_ConA - N_OCT4_WFL	.473	.103	.727	.019
	N_OCT4_AAL - N_OCT4_UEA	.029	-.296	.349	.861
	N_OCT4_AAL - N_OCT4_MAA	.579	.315	.760	<.001
	N_OCT4_AAL - N_OCT4_SBA	.229	-.113	.522	.185
	N_OCT4_AAL - N_OCT4_WFL	.143	-.242	.489	.458
	N_OCT4_UEA - N_OCT4_MAA	.193	-.138	.485	.248
	N_OCT4_UEA - N_OCT4_SBA	.020	-.316	.350	.910
	N_OCT4_UEA - N_OCT4_WFL	.067	-.313	.428	.729
	N_OCT4_MAA - N_OCT4_SBA	.438	.123	.673	.011
	N_OCT4_MAA - N_OCT4_WFL	.314	-.065	.614	.102
	N_OCT4_SBA - N_OCT4_WFL	.314	-.065	.614	.102
**MET:**	MET_ConA - MET_AAL	.553	.328	.719	<.001
	MET_ConA - MET_UEA	.493	.245	.680	<.001
	MET_ConA - MET_MAA	.385	.101	.612	.012
	MET_ConA - MET_SBA	.463	.194	.668	.003
	MET_ConA - MET_WFL	.509	.223	.714	.002
	MET_ConA - N_MET_ConA	-.072	-.395	.268	.677
	MET_ConA - N_MET_AAL	-.150	-.460	.192	.384
	MET_ConA - N_MET_UEA	.072	-.268	.395	.677
	MET_ConA - N_MET_MAA	-.124	-.439	.218	.472
	MET_ConA - N_MET_SBA	-.162	-.480	.194	.365
	MET_ConA - N_MET_WFL	.100	-.267	.442	.589
	MET_AAL - MET_UEA	.367	.101	.583	.010
	MET_AAL - MET_MAA	.502	.249	.691	<.001
	MET_AAL - MET_SBA	.542	.300	.718	<.001
	MET_AAL - MET_WFL	.516	.242	.714	.001
	MET_AAL - N_MET_ConA	-.255	-.542	.085	.140
	MET_AAL - N_MET_AAL	-.006	-.328	.317	.972
	MET_AAL - N_MET_UEA	-.041	-.359	.285	.807
	MET_AAL - N_MET_MAA	-.123	-.428	.208	.463
	MET_AAL - N_MET_SBA	-.242	-.532	.099	.161
	MET_AAL - N_MET_WFL	-.250	-.547	.104	.161
	MET_UEA - MET_MAA	.407	.134	.623	.007
	MET_UEA - MET_SBA	.336	.052	.570	.025
	MET_UEA - MET_WFL	.411	.112	.641	.011
	MET_UEA - N_MET_ConA	.059	-.292	.395	.742
	MET_UEA - N_MET_AAL	-.059	-.384	.280	.733
	MET_UEA - N_MET_UEA	.216	-.126	.512	.211
	MET_UEA - N_MET_MAA	-.176	-.481	.166	.306
	MET_UEA - N_MET_SBA	-.015	-.358	.332	.934
	MET_UEA - N_MET_WFL	.167	-.203	.495	.368
	MET_MAA - MET_SBA	.749	.582	.855	<.001
	MET_MAA - MET_WFL	.591	.331	.767	<.001
	MET_MAA - N_MET_ConA	-.083	-.428	.283	.653
	MET_MAA - N_MET_AAL	-.132	-.456	.222	.458
	MET_MAA - N_MET_UEA	-.088	-.420	.264	.621
	MET_MAA - N_MET_MAA	-.162	-.480	.194	.365
	MET_MAA - N_MET_SBA	-.033	-.386	.328	.857
	MET_MAA - N_MET_WFL	-.143	-.489	.242	.458
	MET_SBA - MET_WFL	.611	.368	.775	<.001
	MET_SBA - N_MET_ConA	-.167	-.495	.203	.368
	MET_SBA - N_MET_AAL	-.118	-.444	.237	.510
	MET_SBA - N_MET_UEA	-.074	-.408	.278	.680
	MET_SBA - N_MET_MAA	-.029	-.370	.318	.869
	MET_SBA - N_MET_SBA	.017	-.343	.372	.928
	MET_SBA - N_MET_WFL	-.276	-.588	.107	.151
	MET_WFL - N_MET_ConA	.154	-.270	.528	.464
	MET_WFL - N_MET_AAL	-.077	-.452	.321	.702
	MET_WFL - N_MET_UEA	.121	-.281	.486	.547
	MET_WFL - N_MET_MAA	-.077	-.452	.321	.702
	MET_WFL - N_MET_SBA	.128	-.294	.509	.542
	MET_WFL - N_MET_WFL	.103	-.318	.489	.625
	N_MET_ConA - N_MET_AAL	.294	-.043	.571	.088
	N_MET_ConA - N_MET_UEA	.281	-.057	.562	.103
	N_MET_ConA - N_MET_MAA	.425	.107	.664	.014
	N_MET_ConA - N_MET_SBA	.485	.169	.711	.007
	N_MET_ConA - N_MET_WFL	.533	.217	.748	.004
	N_MET_AAL - N_MET_UEA	.146	-.185	.448	.382
	N_MET_AAL - N_MET_MAA	.509	.223	.714	.002
	N_MET_AAL - N_MET_SBA	.412	.091	.655	.017
	N_MET_AAL - N_MET_WFL	.059	-.292	.395	.742
	N_MET_UEA - N_MET_MAA	.310	-.014	.575	.064
	N_MET_UEA - N_MET_SBA	.229	-.113	.522	.185
	N_MET_UEA - N_MET_WFL	.426	.096	.672	.017
	N_MET_MAA - N_MET_SBA	.621	.363	.791	<.001
	N_MET_MAA - N_MET_WFL	.338	-.007	.612	.058
	N_MET_SBA - N_MET_WFL	.426	.096	.672	.017
**CIP2A:**	CIP2A_ConA - CIP2A_AAL	.473	.228	.662	<.001
	CIP2A_ConA - CIP2A_UEA	.420	.157	.628	.004
	CIP2A_ConA - CIP2A_MAA	.515	.258	.704	<.001
	CIP2A_ConA - CIP2A_SBA	.463	.194	.668	.003
	CIP2A_ConA - CIP2A_WFL	.520	.238	.722	.002
	CIP2A_ConA - N_CIP2A_ConA	.203	-.140	.502	.240
	CIP2A_ConA - N_CIP2A_AAL	.046	-.292	.373	.791
	CIP2A_ConA - N_CIP2A_UEA	.111	-.230	.428	.520
	CIP2A_ConA - N_CIP2A_MAA	-.137	-.450	.205	.426
	CIP2A_ConA - N_CIP2A_SBA	-.214	-.520	.141	.232
	CIP2A_ConA - N_CIP2A_WFL	.100	-.267	.442	.589
	CIP2A_AAL - CIP2A_UEA	.373	.109	.588	.009
	CIP2A_AAL - CIP2A_MAA	.399	.125	.617	.008
	CIP2A_AAL - CIP2A_SBA	.494	.239	.685	<.001
	CIP2A_AAL - CIP2A_WFL	.411	.112	.641	.011
	CIP2A_AAL - N_CIP2A_ConA	.006	-.317	.328	.972
	CIP2A_AAL - N_CIP2A_AAL	.018	-.307	.338	.916
	CIP2A_AAL - N_CIP2A_UEA	-.006	-.328	.317	.972
	CIP2A_AAL - N_CIP2A_MAA	-.076	-.389	.253	.649
	CIP2A_AAL - N_CIP2A_SBA	-.223	-.518	.119	.197
	CIP2A_AAL - N_CIP2A_WFL	-.147	-.468	.208	.410
	CIP2A_UEA - CIP2A_MAA	.312	.026	.551	.037
	CIP2A_UEA - CIP2A_SBA	.249	-.043	.502	.096
	CIP2A_UEA - CIP2A_WFL	.411	.112	.641	.011
	CIP2A_UEA - N_CIP2A_ConA	.085	-.255	.407	.622
	CIP2A_UEA - N_CIP2A_AAL	.072	-.268	.395	.677
	CIP2A_SBA - N_LIMA1_SBA	-.133	-.469	.235	.471
	CIP2A_SBA - N_OCT4_SBA	-.117	-.455	.251	.528
	CIP2A_SBA - N_MET_SBA	.000	-.358	.358	1.000
	N_LIMA1_SBA - N_OCT4_SBA	.647	.400	.806	<.001
	N_LIMA1_SBA - N_MET_SBA	.529	.238	.733	.002
	N_LIMA1_SBA - N_CIP2A_SBA	.577	.302	.763	<.001
	N_OCT4_SBA - N_MET_SBA	.595	.326	.774	<.001
	N_OCT4_SBA - N_CIP2A_SBA	.564	.284	.755	.001
	N_MET_SBA - N_CIP2A_SBA	.485	.182	.704	.005
**WFL:**	LIMA1_WFL - OCT4_WFL	.595	.326	.774	<.001
	LIMA1_WFL - MET_WFL	.653	.427	.802	<.001
	LIMA1_WFL - CIP2A_WFL	.474	.189	.685	.004
	OCT4_WFL - MET_WFL	.869	.755	.932	<.001
	OCT4_WFL - CIP2A_WFL	.673	.438	.822	<.001
	MET_WFL - CIP2A_WFL	.695	.488	.828	<.001
	LIMA1_WFL - N_OCT4_WFL	.051	-.363	.449	.807
	LIMA1_WFL - N_MET_WFL	.000	-.407	.407	1.000
	LIMA1_WFL - N_CIP2A_WFL	.026	-.385	.428	.903
	OCT4_WFL - N_LIMA1_WFL	.154	-.270	.528	.464
	OCT4_WFL - N_MET_WFL	.154	-.270	.528	.464
	OCT4_WFL - N_CIP2A_WFL	.179	-.245	.546	.393
	MET_WFL - N_LIMA1_WFL	.103	-.318	.489	.625
	MET_WFL - N_OCT4_WFL	.000	-.407	.407	1.000
	MET_WFL - N_CIP2A_WFL	.231	-.194	.583	.272
	CIP2A_WFL - N_LIMA1_WFL	.103	-.318	.489	.625
	CIP2A_WFL - N_OCT4_WFL	-.154	-.528	.270	.464
	CIP2A_WFL - N_MET_WFL	.103	-.318	.489	.625
	N_LIMA1_WFL - N_OCT4_WFL	.486	.139	.726	.012
	N_LIMA1_WFL - N_MET_WFL	.574	.285	.767	.001
	N_LIMA1_WFL - N_CIP2A_WFL	.368	.026	.632	.039
	N_OCT4_WFL - N_MET_WFL	.600	.294	.795	.002
	N_OCT4_WFL - N_CIP2A_WFL	.467	.115	.715	.015
	N_MET_WFL - N_CIP2A_WFL	.735	.523	.862	<.001

	CIP2A_UEA - N_CIP2A_UEA	.059	-.280	.384	.733
	CIP2A_UEA - N_CIP2A_MAA	-.255	-.542	.085	.140
	CIP2A_UEA - N_CIP2A_SBA	-.022	-.364	.325	.902
	CIP2A_UEA - N_CIP2A_WFL	.083	-.283	.428	.653
	CIP2A_MAA - CIP2A_SBA	.567	.326	.739	<.001
	CIP2A_MAA - CIP2A_WFL	.345	.025	.601	.039
	CIP2A_MAA - N_CIP2A_ConA	.118	-.237	.444	.510
	CIP2A_MAA - N_CIP2A_AAL	-.059	-.395	.292	.742
	CIP2A_MAA - N_CIP2A_UEA	.044	-.305	.383	.805
	CIP2A_MAA - N_CIP2A_MAA	-.309	-.591	.040	.084
	CIP2A_MAA - N_CIP2A_SBA	-.333	-.617	.027	.072
	CIP2A_MAA - N_CIP2A_WFL	.010	-.364	.380	.961
	CIP2A_SBA - CIP2A_WFL	.421	.124	.649	.009
	CIP2A_SBA - N_CIP2A_ConA	.206	-.149	.514	.249
	CIP2A_SBA - N_CIP2A_AAL	-.147	-.468	.208	.410
	CIP2A_SBA - N_CIP2A_UEA	-.015	-.358	.332	.934
	CIP2A_SBA - N_CIP2A_MAA	-.132	-.456	.222	.458
	CIP2A_SBA - N_CIP2A_SBA	-.283	-.582	.083	.126
	CIP2A_SBA - N_CIP2A_WFL	.181	-.205	.518	.347
	CIP2A_WFL - N_CIP2A_ConA	.077	-.321	.452	.702
	CIP2A_WFL - N_CIP2A_AAL	-.055	-.434	.341	.784
	CIP2A_WFL - N_CIP2A_UEA	.077	-.321	.452	.702
	CIP2A_WFL - N_CIP2A_MAA	.011	-.379	.398	.956
	CIP2A_WFL - N_CIP2A_SBA	-.051	-.449	.363	.807
	CIP2A_WFL - N_CIP2A_WFL	.179	-.245	.546	.393
	N_CIP2A_ConA - N_CIP2A_AAL	.193	-.138	.485	.248
	N_CIP2A_ConA - N_CIP2A_UEA	.170	-.162	.467	.310
	N_CIP2A_ConA - N_CIP2A_MAA	.076	-.253	.389	.649
	N_CIP2A_ConA - N_CIP2A_SBA	.446	.133	.678	.010
	N_CIP2A_ConA - N_CIP2A_WFL	.471	.150	.701	.008
	N_CIP2A_AAL - N_CIP2A_UEA	.111	-.219	.419	.506
	N_CIP2A_AAL - N_CIP2A_MAA	.135	-.197	.438	.421
	N_CIP2A_AAL - N_CIP2A_SBA	.223	-.119	.518	.197
	N_CIP2A_AAL - N_CIP2A_WFL	-.132	-.456	.222	.458
	N_CIP2A_UEA - N_CIP2A_MAA	.041	-.285	.359	.807
	N_CIP2A_UEA - N_CIP2A_SBA	.118	-.224	.434	.495
	N_CIP2A_UEA - N_CIP2A_WFL	.162	-.194	.480	.365
	N_CIP2A_MAA - N_CIP2A_SBA	.393	.069	.642	.023
	N_CIP2A_MAA - N_CIP2A_WFL	.118	-.237	.444	.510
	N_CIP2A_SBA - N_CIP2A_WFL	.376	.036	.638	.035
