# Supplementary material for: Identification of stemness-related glycosylation changes in head and neck squamous cell carcinoma
Source: BMC Cancer. 2024 Apr 10;24:443. doi: 10.1186/s12885-024-12161-5 (PMC11005150; doi:10.1186/s12885-024-12161-5)
Supplement: Supplementary file 4 — Supplementary Material 4 [file 12885_2024_12161_MOESM4_ESM.pdf]

Supplemental Table 3. **Association with TNM factors, recidive, radiotherapy response, alcohol and tobacco use, comorbidities, sex and age in tumor tissue, adjacent normal tissue (N) and serum (S).**

Nodal positivity (N+)

	γ (Hedges)	95% Confidence Interval Lower	Upper	p
LIMA1_ConA	.035	-.815	.884	.937
LIMA1_AAL	.009	-.804	.821	.984
LIMA1_UEA	-.478	-1.334	.388	.282
LIMA1_MAA	-.289	-1.192	.622	.538
LIMA1_SBA	.243	-.619	1.099	.584
LIMA1_WFL	-.556	-1.447	.350	.373
OCT4_ConA	.223	-.632	1.073	.612
OCT4_AAL	.400	-.425	1.217	.344
OCT4_UEA	-.627	-1.537	.297	.449
OCT4_MAA	-.348	-1.253	.565	.458
OCT4_SBA	-.268	-1.171	.642	.568
OCT4_WFL	.042	-.892	.975	.930
MET_ConA	.178	-.675	1.027	.685
MET_AAL	.009	-.804	.821	.983
MET_UEA	-.860	-1.738	.035	.249
MET_MAA	-.109	-1.010	.796	.816
MET_SBA	-.114	-.969	.744	.797
MET_WFL	-.053	-.933	.827	.907
CIP2A_ConA	-.079	-.928	.772	.857
CIP2A_AAL	.040	-.773	.852	.924
CIP2A_UEA	-.771	-1.642	.117	.249
CIP2A_MAA	.173	-.733	1.075	.711
CIP2A_SBA	-.076	-.931	.782	.864
CIP2A_WFL	.280	-.608	1.161	.541
N_LIMA1_ConA	.137	-.790	1.060	.774
N_LIMA1_AAL	.209	-.721	1.132	.664
N_LIMA1_UEA	.395	-.544	1.322	.414
N_LIMA1_MAA	.252	-.679	1.176	.600
N_LIMA1_SBA	.303	-.641	1.237	.534
N_LIMA1_WFL	.466	-.500	1.416	.224
N_OCT4_ConA	-.025	-1.007	.958	.961
N_OCT4_AAL	.074	-.852	.997	.878
N_OCT4_UEA	-.258	-1.182	.673	.591
N_OCT4_MAA	-.381	-1.308	.557	.430
N_OCT4_SBA	-.284	-1.218	.659	.560
N_OCT4_WFL	-.037	-1.008	.936	.942
N_MET_ConA	.241	-.749	1.222	.638
N_MET_AAL	.207	-.723	1.130	.666
N_MET_UEA	.343	-.592	1.270	.476
N_MET_MAA	.305	-.629	1.230	.526
N_MET_SBA	.282	-.661	1.216	.562
N_MET_WFL	.442	-.522	1.392	.374
N_CIP2A_ConA	.262	-.670	1.186	.586
N_CIP2A_AAL	-.359	-1.286	.577	.456
N_CIP2A_UEA	-.021	-.944	.903	.965
N_CIP2A_MAA	-.100	-1.023	.826	.835
N_CIP2A_SBA	-.548	-1.492	.412	.266
N_CIP2A_WFL	.495	-.473	1.447	.321
S_LIMA1_ConA	.386	-.544	1.305	.420
S_LIMA1_AAL	1.154	.014	2.255	.016
S_LIMA1_UEA	.598	-.346	1.526	.217
S_LIMA1_MAA	.970	-.542	2.423	.215
S_LIMA1_SBA	.824	-.140	1.768	.095
S_LIMA1_WFL	-.537	-1.462	.402	.265
S_OCT4_ConA	-.496	-1.898	.940	.762
S_OCT4_AAL	1.161	-.800	3.025	.
S_OCT4_UEA	.303	-1.112	1.697	.683
S_OCT4_MAA	-.096	-1.489	1.304	.897
S_OCT4_SBA	.800	-.681	2.229	.299
S_OCT4_WFL	.664	-1.262	2.559	.
S_MET_ConA	.453	-.481	1.374	.345
S_MET_AAL	1.253	.095	2.369	.011
S_MET_UEA	1.259	.241	2.247	.001
S_MET_MAA	.522	-.917	1.926	.488
S_MET_SBA	.459	-.475	1.381	.339
S_MET_WFL	.353	-.575	1.271	.460
S_CIP2A_ConA	-.214	-1.130	.707	.652
S_CIP2A_AAL	.871	-.225	1.934	.053
S_CIP2A_UEA	.469	-.466	1.391	.195
S_CIP2A_MAA	.614	-.838	2.024	.417
S_CIP2A_SBA	.496	-.441	1.419	.303
S_CIP2A_WFL	.578	-.425	1.564	.262

Distant metastasis (at presentation or during follow-up)

	γ (Hedges)	95% Confidence Interval Lower	Upper	p
LIMA1_ConA	-.699	-2.104	.721	.338
LIMA1_AAL	-.391	-1.559	.785	.518
LIMA1_UEA	-1.070	-2.268	.151	.434
LIMA1_MAA	.403	-1.005	1.802	.578
LIMA1_SBA	.380	-.799	1.551	.531
LIMA1_WFL	-.925	-2.126	.300	.531
OCT4_ConA	-.492	-1.891	.919	.498
OCT4_AAL	.265	-.907	1.431	.661
OCT4_UEA	.150	-1.250	1.547	.835
OCT4_MAA	.371	-1.037	1.769	.609
OCT4_SBA	.452	-.959	1.851	.534
OCT4_WFL	.528	-.892	1.931	.471
MET_ConA	-.408	-1.806	1.000	.573
MET_AAL	-.250	-1.416	.922	.679
MET_UEA	-1.931	-3.212	-.615	.357
MET_MAA	.427	-.983	1.826	.556
MET_SBA	.488	-.697	1.661	.423
MET_WFL	-.082	-1.257	1.095	.893
CIP2A_ConA	-.638	-2.041	.779	.381
CIP2A_AAL	.314	-.859	1.481	.602
CIP2A_UEA	-1.266	-2.478	-.027	.414
CIP2A_MAA	.486	-.926	1.886	.504
CIP2A_SBA	.452	-.730	1.624	.457
CIP2A_WFL	.214	-.967	1.388	.726
N_LIMA1_ConA	.240	-1.165	1.638	.741
N_LIMA1_AAL	.220	-1.184	1.619	.761
N_LIMA1_UEA	.177	-1.226	1.576	.807
N_LIMA1_MAA	.354	-1.056	1.753	.627
N_LIMA1_SBA	.214	-1.191	1.613	.768
N_LIMA1_WFL	.188	-1.217	1.587	.796
N_OCT4_ConA	-.197	-2.115	1.728	.844
N_OCT4_AAL	.132	-1.270	1.530	.855
N_OCT4_UEA	-.163	-1.561	1.239	.822
N_OCT4_MAA	-.277	-1.675	1.129	.294
N_OCT4_SBA	-.328	-1.728	1.081	.652
N_OCT4_WFL	-1.290	-2.752	.217	.094
N_MET_ConA	.296	-1.632	2.215	.766
N_MET_AAL	.225	-1.179	1.624	.756
N_MET_UEA	.160	-1.243	1.558	.825
N_MET_MAA	.376	-1.035	1.775	.605
N_MET_SBA	.432	-.982	1.833	.554
N_MET_WFL	-.101	-1.500	1.301	.889
N_CIP2A_ConA	.140	-1.262	1.538	.847
N_CIP2A_AAL	-.073	-1.471	1.328	.920
N_CIP2A_UEA	-.343	-1.742	1.066	.637
N_CIP2A_MAA	.983	-.468	2.407	.186
N_CIP2A_SBA	-.007	-1.407	1.393	.992
N_CIP2A_WFL	.207	-1.198	1.606	.776
S_LIMA1_ConA	.860	-.578	2.275	.244
S_LIMA1_AAL	.763	-.685	2.182	.056
S_LIMA1_UEA	.466	-.947	1.867	.522
S_LIMA1_MAA	.	.	.	.
S_LIMA1_SBA	1.139	-.323	2.571	.128
S_LIMA1_WFL	-.472	-1.872	.942	.517
S_OCT4_ConA	.	.	.	.
S_OCT4_AAL	.	.	.	.
S_OCT4_UEA	.	.	.	.
S_OCT4_MAA	.	.	.	.
S_OCT4_SBA	.	.	.	.
S_OCT4_WFL	.	.	.	.
S_MET_ConA	.943	-.501	2.363	.203
S_MET_AAL	1.014	-.463	2.452	.004
S_MET_UEA	.935	-.509	2.354	.207
S_MET_MAA	.	.	.	.
S_MET_SBA	.374	-1.035	1.773	.607
S_MET_WFL	.208	-1.195	1.606	.774
S_CIP2A_ConA	.163	-1.240	1.560	.822
S_CIP2A_AAL	.733	-.713	2.149	.326
S_CIP2A_UEA	.184	-1.219	1.582	.799
S_CIP2A_MAA	.	.	.	.
S_CIP2A_SBA	.551	-.867	1.954	.450
S_CIP2A_WFL	.239	-1.166	1.637	.742

Recurrence (local, node or distant)

	γ (Hedges)	95% Confidence Interval Lower	Upper	p
LIMA1_ConA	-.003	-.954	.949	.996
LIMA1_AAL	-.145	-1.032	.745	.752
LIMA1_UEA	-.507	-1.405	.403	.278
LIMA1_MAA	.455	-.602	1.501	.402
LIMA1_SBA	.452	-.518	1.411	.364
LIMA1_WFL	-.544	-1.601	.527	.592
OCT4_ConA	.044	-.908	.994	.929
OCT4_AAL	.291	-.603	1.180	.526
OCT4_UEA	-.139	-1.094	.818	.778
OCT4_MAA	.429	-.627	1.474	.430
OCT4_SBA	.508	-.552	1.556	.055
OCT4_WFL	.597	-.610	1.786	.040
MET_ConA	.075	-.877	1.026	.878
MET_AAL	.018	-.870	.905	.969
MET_UEA	-1.114	-2.054	-.153	.206
MET_MAA	.423	-.632	1.468	.436
MET_SBA	.563	-.413	1.526	.040
MET_WFL	.123	-.929	1.171	.822
CIP2A_ConA	.008	-.943	.959	.986
CIP2A_AAL	.377	-.521	1.267	.414
CIP2A_UEA	-.656	-1.562	.264	.163
CIP2A_MAA	.521	-.540	1.569	.050
CIP2A_SBA	.538	-.437	1.500	.049
CIP2A_WFL	.226	-.828	1.275	.677
N_LIMA1_ConA	.452	-.541	1.432	.376
N_LIMA1_AAL	.284	-.700	1.260	.575
N_LIMA1_UEA	.405	-.585	1.384	.427
N_LIMA1_MAA	.304	-.681	1.280	.549
N_LIMA1_SBA	.297	-.695	1.280	.561
N_LIMA1_WFL	-.048	-1.037	.943	.926
N_OCT4_ConA	.256	-.810	1.314	.642
N_OCT4_AAL	.190	-.791	1.164	.708
N_OCT4_UEA	.139	-.839	1.113	.783
N_OCT4_MAA	-.181	-1.155	.799	.721
N_OCT4_SBA	.211	-.777	1.192	.679
N_OCT4_WFL	-.314	-1.391	.776	.578
N_MET_ConA	.409	-.665	1.470	.460
N_MET_AAL	.311	-.674	1.287	.540
N_MET_UEA	.292	-.692	1.268	.565
N_MET_MAA	.301	-.684	1.277	.553
N_MET_SBA	.425	-.575	1.411	.409
N_MET_WFL	-.080	-1.069	.912	.876
N_CIP2A_ConA	.319	-.667	1.295	.530
N_CIP2A_AAL	.167	-.812	1.141	.741
N_CIP2A_UEA	.308	-.678	1.284	.545
N_CIP2A_MAA	.087	-.890	1.062	.863
N_CIP2A_SBA	.040	-.943	1.021	.938
N_CIP2A_WFL	.115	-.878	1.104	.823

High T class (T 1-2 / 3-4)

	γ (Hedges)	95% Confidence Interval Lower	Upper	p
LIMA1_ConA	-.375	-1.176	.435	.367
LIMA1_AAL	-.574	-1.359	.223	.243
LIMA1_UEA	-.900	-1.718	-.064	.089
LIMA1_MAA	-.953	-1.811	-.074	.097
LIMA1_SBA	-.551	-1.356	.267	.249
LIMA1_WFL	-.758	-1.623	.126	.107
OCT4_ConA	-.149	-.946	.652	.719
OCT4_AAL	-.196	-.970	.582	.624
OCT4_UEA	-.454	-1.267	.370	.283
OCT4_MAA	-.773	-1.616	.087	.161
OCT4_SBA	-.789	-1.633	.073	.141
OCT4_WFL	-1.047	-1.984	-.083	.043
MET_ConA	-.258	-1.056	.547	.533
MET_AAL	-.611	-1.398	.189	.135
MET_UEA	-.708	-1.511	.110	.164
MET_MAA	-.845	-1.694	.022	.132
MET_SBA	-.803	-1.625	.036	.105
MET_WFL	-1.031	-1.923	-.115	.034
CIP2A_ConA	-.783	-1.605	.056	.068
CIP2A_AAL	-.043	-.817	.731	.913
CIP2A_UEA	-.899	-1.717	-.063	.085
CIP2A_MAA	-.819	-1.665	.046	.134
CIP2A_SBA	-.306	-1.103	.497	.458
CIP2A_WFL	-.181	-1.020	.663	.678
N_LIMA1_ConA	.144	-.750	1.033	.755
N_LIMA1_AAL	.277	-.622	1.168	.550
N_LIMA1_UEA	.401	-.505	1.296	.390
N_LIMA1_MAA	.362	-.542	1.255	.437
N_LIMA1_SBA	.357	-.559	1.262	.449
N_LIMA1_WFL	.568	-.379	1.498	.180
N_OCT4_ConA	.011	-.922	.944	.982
N_OCT4_AAL	.148	-.746	1.037	.749
N_OCT4_UEA	.010	-.881	.900	.983
N_OCT4_MAA	-.159	-1.048	.735	.731
N_OCT4_SBA	.174	-.733	1.076	.737
N_OCT4_WFL	.062	-.894	1.016	.901
N_MET_ConA	.278	-.665	1.212	.568
N_MET_AAL	.254	-.644	1.145	.583
N_MET_UEA	.516	-.398	1.416	.165
N_MET_MAA	.484	-.427	1.382	.301
N_MET_SBA	.465	-.459	1.374	.328
N_MET_WFL	.627	-.326	1.561	.200
N_CIP2A_ConA	-.661	-1.569	.266	.316
N_CIP2A_AAL	-.160	-1.050	.734	.728
N_CIP2A_UEA	.024	-.867	.914	.959
N_CIP2A_MAA	.698	-.231	1.609	.143
N_CIP2A_SBA	-.219	-1.121	.690	.641
N_CIP2A_WFL	.566	-.382	1.495	.245

Radiotherapy response

	95% Confidence Interval			
	γ (Hedges)	Lower	Upper	p
LIMA1_ConA	.943	-.504	2.364	.204
LIMA1_AAL	.743	-.467	1.933	.602
LIMA1_UEA	1.264	.004	2.494	.476
LIMA1_MAA	-.374	-1.774	1.037	.607
LIMA1_SBA	-.440	-1.620	.753	.474
LIMA1_WFL	1.680	.134	3.178	.535
OCT4_ConA	.444	-.969	1.845	.542
OCT4_AAL	-.238	-1.413	.943	.696
OCT4_UEA	-.355	-1.754	1.055	.626
OCT4_MAA	-.444	-1.845	.971	.543
OCT4_SBA	-.514	-1.917	.905	.482
OCT4_WFL	-.410	-2.322	1.517	.681
MET_ConA	.634	-.791	2.040	.387
MET_AAL	.522	-.673	1.702	.691
MET_UEA	1.539	.244	2.798	.409
MET_MAA	-.455	-1.856	.961	.533
MET_SBA	-.508	-1.690	.689	.410
MET_WFL	.404	-1.010	1.805	.580
CIP2A_ConA	.498	-.918	1.900	.495
CIP2A_AAL	-.333	-1.508	.852	.586
CIP2A_UEA	1.040	-.196	2.250	.514
CIP2A_MAA	-.455	-1.857	.960	.533
CIP2A_SBA	-.445	-1.626	.748	.469
CIP2A_WFL	-.044	-1.444	1.357	.951
N_LIMA1_ConA	-.618	-2.027	.813	.403
N_LIMA1_AAL	-.439	-1.841	.980	.550
N_LIMA1_UEA	-.298	-1.698	1.113	.683
N_LIMA1_MAA	-.402	-1.804	1.015	.583
N_LIMA1_SBA	-.329	-1.729	1.085	.654
N_LIMA1_WFL	.536	-.892	1.942	.468
N_OCT4_ConA	-.287	-2.188	1.626	.773
N_OCT4_AAL	-.654	-2.065	.781	.377
N_OCT4_UEA	.284	-1.127	1.683	.698
N_OCT4_MAA	-.654	-2.065	.781	.377
N_OCT4_SBA	-1.026	-2.465	.452	.177
N_OCT4_WFL	2.331	.146	4.428	.036
N_MET_ConA	-.341	-2.243	1.574	.731
N_MET_AAL	-.422	-1.824	.996	.565
N_MET_UEA	-.106	-1.506	1.297	.884
N_MET_MAA	-.519	-1.924	.905	.481
N_MET_SBA	-.860	-2.285	.599	.252
N_MET_WFL	.455	-.967	1.858	.537
N_CIP2A_ConA	-.404	-1.806	1.012	.581
N_CIP2A_AAL	-.370	-1.771	1.044	.613
N_CIP2A_UEA	.296	-1.115	1.696	.685
N_CIP2A_MAA	-.044	-1.444	1.358	.952
N_CIP2A_SBA	-.176	-1.575	1.231	.810
N_CIP2A_WFL	-.268	-1.667	1.143	.714

Current alcohol use

	95% Confidence Interval			
	γ (Hedges)	Lower	Upper	p
LIMA1_ConA	.108	-1.062	1.275	.858
LIMA1_AAL	.401	-.775	1.569	.507
LIMA1_UEA	.297	-.877	1.465	.623
LIMA1_MAA	.347	-.834	1.519	.568
LIMA1_SBA	.315	-.864	1.487	.604
LIMA1_WFL	.440	-.973	1.841	.546
OCT4_ConA	.177	-.994	1.345	.769
OCT4_AAL	.349	-.826	1.516	.564
OCT4_UEA	.201	-.972	1.370	.739
OCT4_MAA	.326	-.854	1.497	.592
OCT4_SBA	.428	-.759	1.603	.484
OCT4_WFL	.439	-.977	1.841	.548
MET_ConA	-.181	-1.348	.990	.764
MET_AAL	.394	-.782	1.561	.515
MET_UEA	.354	-.823	1.522	.559
MET_MAA	.365	-.816	1.537	.548
MET_SBA	.431	-.753	1.605	.479
MET_WFL	.549	-.870	1.953	.452
CIP2A_ConA	.651	-.540	1.827	.286
CIP2A_AAL	.343	-.831	1.510	.570
CIP2A_UEA	.295	-.880	1.462	.626
CIP2A_MAA	.421	-.762	1.595	.489
CIP2A_SBA	.077	-1.095	1.248	.899
CIP2A_WFL	.513	-.904	1.916	.482
N_LIMA1_ConA	.218	-1.187	1.617	.764
N_LIMA1_AAL	.263	-1.143	1.662	.717
N_LIMA1_UEA	.433	-.981	1.834	.553
N_LIMA1_MAA	.323	-1.086	1.722	.657
N_LIMA1_SBA	.198	-1.207	1.597	.785
N_LIMA1_WFL	.398	-1.528	2.310	.690
N_OCT4_ConA	.389	-1.025	1.789	.595
N_OCT4_AAL	.281	-1.126	1.680	.699
N_OCT4_UEA	.593	-.831	1.998	.419
N_OCT4_MAA	.826	-.614	2.242	.264
N_OCT4_SBA	-.154	-1.553	1.250	.832
N_OCT4_WFL	-.114	-2.016	1.793	.909
N_MET_ConA	.445	-.971	1.847	.543
N_MET_AAL	.261	-1.145	1.660	.719
N_MET_UEA	.470	-.946	1.872	.520
N_MET_MAA	.326	-1.084	1.725	.655
N_MET_SBA	.451	-.966	1.853	.537
N_MET_WFL	.425	-1.503	2.337	.670
N_CIP2A_ConA	-.321	-1.720	1.088	.659
N_CIP2A_AAL	.837	-.605	2.253	.259
N_CIP2A_UEA	.504	-.914	1.907	.490
N_CIP2A_MAA	.785	-.652	2.199	.288
N_CIP2A_SBA	.400	-1.014	1.801	.584
N_CIP2A_WFL	.012	-1.902	1.925	.990
S_LIMA1_ConA	.088	-1.089	1.262	.885
S_LIMA1_AAL	-.375	-1.776	1.043	.611
S_LIMA1_UEA	-.139	-1.313	1.040	.820
S_LIMA1_MAA	-.484	-2.304	1.375	.621
S_LIMA1_SBA	-.822	-2.016	.394	.188
S_LIMA1_WFL	-.195	-1.370	.984	.748
S_OCT4_ConA	.602	-1.272	2.429	.541
S_OCT4_AAL	.505	-1.270	2.222	.594
S_OCT4_UEA	.436	-1.417	2.254	.655
S_OCT4_MAA	-.178	-1.993	1.652	.855
S_OCT4_SBA	-.655	-2.485	1.226	.507
S_OCT4_WFL	-.084	-1.484	1.319	.908
S_MET_ConA	.122	-1.056	1.296	.841
S_MET_AAL	-.197	-1.596	1.211	.788
S_MET_UEA	-.116	-1.291	1.061	.848
S_MET_MAA	.384	-1.464	2.201	.693
S_MET_SBA	-.768	-1.959	.444	.217
S_MET_WFL	-1.205	-2.428	.049	.430
S_CIP2A_ConA	-.049	-1.224	1.127	.936
S_CIP2A_AAL	.167	-1.240	1.566	.820
S_CIP2A_UEA	-.486	-1.665	.707	.429
S_CIP2A_MAA	-1.060	-2.933	.891	.297
S_CIP2A_SBA	-1.772	-3.060	-.444	.321
S_CIP2A_WFL	-.792	-1.988	.426	.205

Current tobacco use

	95% Confidence Interval			
	γ (Hedges)	Lower	Upper	p
LIMA1_ConA	.758	-.044	1.545	.048
LIMA1_AAL	.842	.050	1.617	.032
LIMA1_UEA	.698	-.094	1.476	.084
LIMA1_MAA	.803	-.031	1.618	.086
LIMA1_SBA	.754	-.075	1.566	.076
LIMA1_WFL	.868	-.028	1.743	.071
OCT4_ConA	.556	-.230	1.330	.131
OCT4_AAL	.622	-.151	1.383	.100
OCT4_UEA	.592	-.206	1.378	.161
OCT4_MAA	.649	-.171	1.454	.153
OCT4_SBA	.456	-.365	1.267	.279
OCT4_WFL	.449	-.451	1.335	.332
MET_ConA	.457	-.323	1.227	.253
MET_AAL	.865	.071	1.642	.028
MET_UEA	.579	-.203	1.350	.144
MET_MAA	.666	-.155	1.472	.142
MET_SBA	.510	-.299	1.307	.219
MET_WFL	.495	-.366	1.343	.263
CIP2A_ConA	.635	-.157	1.413	.117
CIP2A_AAL	.569	-.200	1.327	.130
CIP2A_UEA	.889	.080	1.681	.033
CIP2A_MAA	.967	.116	1.797	.045
CIP2A_SBA	.880	.039	1.702	.043
CIP2A_WFL	.497	-.365	1.345	.261
N_LIMA1_ConA	.145	-.729	1.014	.748
N_LIMA1_AAL	.281	-.598	1.152	.535
N_LIMA1_UEA	.079	-.792	.948	.861
N_LIMA1_MAA	.364	-.519	1.237	.423
N_LIMA1_SBA	.309	-.604	1.213	.512
N_LIMA1_WFL	.188	-.734	1.104	.693
N_OCT4_ConA	-.111	-1.012	.794	.813
N_OCT4_AAL	.210	-.665	1.080	.642
N_OCT4_UEA	.020	-.850	.890	.964
N_OCT4_MAA	-.056	-.925	.815	.901
N_OCT4_SBA	-.786	-1.716	.167	.107
N_OCT4_WFL	-.543	-1.527	.461	.293
N_MET_ConA	.174	-.733	1.075	.711
N_MET_AAL	.269	-.609	1.140	.552
N_MET_UEA	.325	-.556	1.197	.474
N_MET_MAA	.339	-.543	1.212	.455
N_MET_SBA	.169	-.738	1.071	.718
N_MET_WFL	.138	-.782	1.054	.772
N_CIP2A_ConA	.297	-.583	1.168	.512
N_CIP2A_AAL	-.128	-.997	.745	.777
N_CIP2A_UEA	-.352	-1.225	.531	.439
N_CIP2A_MAA	.064	-.807	.934	.886
N_CIP2A_SBA	-.322	-1.226	.592	.494
N_CIP2A_WFL	.295	-.633	1.213	.538
S_LIMA1_ConA	-.353	-1.186	.488	.414
S_LIMA1_AAL	-.079	-1.102	.946	.882
S_LIMA1_UEA	.643	-.219	1.489	.145
S_LIMA1_MAA	1.266	-.104	2.569	.071
S_LIMA1_SBA	.082	-.749	.911	.849
S_LIMA1_WFL	.794	-.082	1.651	.057
S_OCT4_ConA	-.656	-1.851	.580	.277
S_OCT4_AAL	.589	-.745	1.871	.401
S_OCT4_UEA	.495	-.716	1.674	.513
S_OCT4_MAA	.308	-.881	1.476	.622
S_OCT4_SBA	.492	-.719	1.670	.436
S_OCT4_WFL	.556	-.496	1.583	.286
S_MET_ConA	-.254	-1.084	.583	.556
S_MET_AAL	.043	-.982	1.065	.936
S_MET_UEA	.139	-.693	.968	.746
S_MET_MAA	1.089	-.237	2.353	.197
S_MET_SBA	-.275	-1.105	.563	.524
S_MET_WFL	.507	-.345	1.345	.246
S_CIP2A_ConA	-.224	-1.053	.612	.603
S_CIP2A_AAL	.078	-.947	1.100	.884
S_CIP2A_UEA	.211	-.624	1.041	.623
S_CIP2A_MAA	.366	-.829	1.536	.558
S_CIP2A_SBA	.244	-.592	1.075	.570
S_CIP2A_WFL	.327	-.540	1.186	.464

Tobacco history (over 20py)

	95% Confidence Interval			
	γ (Hedges)	Lower	Upper	p
LIMA1_ConA	.025	-.765	.814	.952
LIMA1_AAL	.189	-.597	.971	.640
LIMA1_UEA	.157	-.659	.969	.709
LIMA1_MAA	-.476	-1.379	.438	.095
LIMA1_SBA	-.487	-1.350	.387	.109
LIMA1_WFL	.320	-.660	1.290	.526
OCT4_ConA	-.139	-.929	.653	.732
OCT4_AAL	-.303	-1.087	.487	.456
OCT4_UEA	-.183	-1.032	.671	.677
OCT4_MAA	-.450	-1.352	.462	.337
OCT4_SBA	-.589	-1.503	.338	.051
OCT4_WFL	-.749	-1.827	.351	.020
MET_ConA	.106	-.685	.895	.794
MET_AAL	.208	-.579	.991	.607
MET_UEA	.474	-.354	1.293	.445
MET_MAA	-.448	-1.350	.464	.339
MET_SBA	-.618	-1.487	.265	.047
MET_WFL	-.292	-1.262	.686	.562
CIP2A_ConA	-.277	-1.068	.519	.498
CIP2A_AAL	-.441	-1.229	.356	.145
CIP2A_UEA	.204	-.613	1.017	.627
CIP2A_MAA	-.510	-1.414	.406	.077
CIP2A_SBA	-.413	-1.273	.457	.194
CIP2A_WFL	-.441	-1.415	.545	.385
N_LIMA1_ConA	.335	-.600	1.261	.640
N_LIMA1_AAL	.731	-.235	1.676	.349
N_LIMA1_UEA	.380	-.558	1.307	.590
N_LIMA1_MAA	.738	-.228	1.684	.337
N_LIMA1_SBA	.659	-.311	1.609	.385
N_LIMA1_WFL	.947	-.112	1.979	.247
N_OCT4_ConA	.314	-.630	1.249	.519
N_OCT4_AAL	.792	-.179	1.743	.300
N_OCT4_UEA	.170	-.758	1.094	.722
N_OCT4_MAA	-.059	-.983	.866	.921
N_OCT4_SBA	-.534	-1.477	.425	.203
N_OCT4_WFL	.059	-1.019	1.135	.916
N_MET_ConA	.451	-.502	1.390	.535
N_MET_AAL	.676	-.284	1.618	.383
N_MET_UEA	.433	-.509	1.361	.372
N_MET_MAA	.487	-.458	1.419	.316
N_MET_SBA	-.198	-1.130	.741	.684
N_MET_WFL	.334	-.668	1.326	.518
N_CIP2A_ConA	-.388	-1.316	.550	.422
N_CIP2A_AAL	.460	-.483	1.390	.343
N_CIP2A_UEA	-.262	-1.186	.670	.586
N_CIP2A_MAA	.627	-.329	1.566	.201
N_CIP2A_SBA	-.289	-1.223	.654	.552
N_CIP2A_WFL	-.346	-1.338	.658	.504
S_LIMA1_ConA	.178	-.697	1.049	.693
S_LIMA1_AAL	.673	-.398	1.717	.222
S_LIMA1_UEA	.528	-.366	1.408	.250
S_LIMA1_MAA	.829	-.499	2.106	.228
S_LIMA1_SBA	.451	-.437	1.328	.323
S_LIMA1_WFL	.517	-.376	1.396	.259
S_OCT4_ConA	-.326	-1.557	.928	.487
S_OCT4_AAL	1.279	-.240	2.711	.103
S_OCT4_UEA	.905	-.439	2.193	.433
S_OCT4_MAA	.879	-.459	2.164	.204
S_OCT4_SBA	1.047	-.327	2.360	.140
S_OCT4_WFL	.398	-.663	1.441	.469
S_MET_ConA	.211	-.665	1.082	.640
S_MET_AAL	.712	-.363	1.759	.280
S_MET_UEA	.375	-.508	1.249	.409
S_MET_MAA	2.440	.645	4.156	.100
S_MET_SBA	.320	-.561	1.193	.480
S_MET_WFL	.482	-.409	1.360	.292
S_CIP2A_ConA	-.300	-1.172	.580	.507
S_CIP2A_AAL	.543	-.513	1.578	.454
S_CIP2A_UEA	-.004	-.874	.867	.994
S_CIP2A_MAA	.611	-.680	1.861	.363
S_CIP2A_SBA	.720	-.189	1.611	.281
S_CIP2A_WFL	.082	-.799	.961	.857

Alcohol history

		95% Confidence Interval		
	γ (Hedges)	Lower	Upper	p
LIMA1_ConA	.491	-.300	1.273	.166
LIMA1_AAL	.513	-.271	1.286	.126
LIMA1_UEA	.312	-.470	1.087	.438
LIMA1_MAA	-.445	-1.245	.366	.341
LIMA1_SBA	.195	-.604	.989	.635
LIMA1_WFL	.193	-.656	1.036	.660
OCT4_ConA	.418	-.369	1.197	.300
OCT4_AAL	.441	-.338	1.212	.173
OCT4_UEA	-.404	-1.192	.393	.324
OCT4_MAA	-.694	-1.508	.135	.167
OCT4_SBA	-.602	-1.424	.233	.203
OCT4_WFL	-.466	-1.353	.435	.321
MET_ConA	.181	-.596	.955	.650
MET_AAL	.378	-.398	1.146	.259
MET_UEA	.205	-.573	.979	.608
MET_MAA	-.695	-1.509	.134	.168
MET_SBA	-.591	-1.398	.230	.217
MET_WFL	-.300	-1.145	.553	.521
CIP2A_ConA	.077	-.698	.850	.848
CIP2A_AAL	.140	-.628	.905	.723
CIP2A_UEA	.188	-.590	.961	.639
CIP2A_MAA	-.187	-.981	.612	.650
CIP2A_SBA	-.326	-1.123	.478	.430
CIP2A_WFL	.015	-.829	.858	.973
N_LIMA1_ConA	.007	-.863	.877	.988
N_LIMA1_AAL	.443	-.445	1.319	.332
N_LIMA1_UEA	.139	-.734	1.008	.758
N_LIMA1_MAA	.425	-.462	1.300	.352
N_LIMA1_SBA	.285	-.627	1.188	.545
N_LIMA1_WFL	.607	-.371	1.565	.117
N_OCT4_ConA	.222	-.670	1.108	.629
N_OCT4_AAL	.415	-.472	1.289	.299
N_OCT4_UEA	-.044	-.913	.827	.923
N_OCT4_MAA	.344	-.538	1.216	.449
N_OCT4_SBA	-.399	-1.306	.519	.459
N_OCT4_WFL	-.174	-1.145	.804	.732
N_MET_ConA	.302	-.594	1.188	.514
N_MET_AAL	.445	-.444	1.321	.330
N_MET_UEA	.409	-.478	1.283	.370
N_MET_MAA	.511	-.383	1.390	.205
N_MET_SBA	.287	-.625	1.190	.542
N_MET_WFL	.534	-.437	1.489	.180
N_CIP2A_ConA	-.610	-1.495	.292	.282
N_CIP2A_AAL	.457	-.433	1.334	.264
N_CIP2A_UEA	-.433	-1.309	.455	.343
N_CIP2A_MAA	.239	-.638	1.109	.597
N_CIP2A_SBA	-.401	-1.308	.518	.396
N_CIP2A_WFL	.065	-.881	1.008	.895
S_LIMA1_ConA	.088	-.759	.933	.840
S_LIMA1_AAL	-.167	-1.156	.830	.747
S_LIMA1_UEA	-.019	-.864	.827	.965
S_LIMA1_MAA	.159	-1.017	1.324	.797
S_LIMA1_SBA	-.118	-.962	.730	.788
S_LIMA1_WFL	.198	-.653	1.043	.652
S_OCT4_ConA	1.201	-.152	2.490	.075
S_OCT4_AAL	1.279	-.240	2.711	.103
S_OCT4_UEA	.423	-.779	1.596	.501
S_OCT4_MAA	.941	-.351	2.177	.158
S_OCT4_SBA	.442	-.763	1.616	.483
S_OCT4_WFL	.546	-.505	1.573	.314
S_MET_ConA	.079	-.768	.924	.857
S_MET_AAL	-.056	-1.045	.936	.914
S_MET_UEA	-.149	-.994	.700	.734
S_MET_MAA	.722	-.526	1.924	.238
S_MET_SBA	-.075	-.920	.772	.864
S_MET_WFL	-.205	-1.050	.646	.703
S_CIP2A_ConA	.198	-.653	1.043	.652
S_CIP2A_AAL	.112	-.882	1.101	.828
S_CIP2A_UEA	-.296	-1.143	.558	.500
S_CIP2A_MAA	.255	-.929	1.421	.682
S_CIP2A_SBA	-.603	-1.462	.271	.178
S_CIP2A_WFL	-.092	-.948	.766	.835

Cardiovascular disease (hypertension, coronary artery disease)

		95% Confidence Interval		
	γ (Hedges)	Lower	Upper	p
LIMA1_ConA	-.314	-1.128	.507	.457
LIMA1_AAL	-.336	-1.120	.456	.409
LIMA1_UEA	-.364	-1.157	.436	.252
LIMA1_MAA	-.050	-.876	.778	.907
LIMA1_SBA	-.302	-1.160	.562	.496
LIMA1_WFL	-.402	-1.322	.529	.401
OCT4_ConA	-.433	-1.251	.393	.307
OCT4_AAL	-.396	-1.183	.398	.331
OCT4_UEA	-.046	-.843	.753	.912
OCT4_MAA	.004	-.823	.831	.993
OCT4_SBA	-.294	-1.158	.578	.512
OCT4_WFL	-.396	-1.333	.553	.418
MET_ConA	-.184	-.996	.632	.661
MET_AAL	-.590	-1.384	.216	.073
MET_UEA	-.127	-.916	.665	.756
MET_MAA	.172	-.659	.998	.688
MET_SBA	-.259	-1.115	.604	.560
MET_WFL	-.486	-1.409	.450	.312
CIP2A_ConA	-.428	-1.245	.399	.313
CIP2A_AAL	-.446	-1.234	.351	.141
CIP2A_UEA	-.459	-1.255	.346	.160
CIP2A_MAA	-.005	-.832	.822	.991
CIP2A_SBA	-.179	-1.034	.681	.687
CIP2A_WFL	-.394	-1.313	.536	.410
N_LIMA1_ConA	-.355	-1.248	.548	.445
N_LIMA1_AAL	-.368	-1.262	.536	.429
N_LIMA1_UEA	-.518	-1.418	.396	.270
N_LIMA1_MAA	-.382	-1.276	.523	.412
N_LIMA1_SBA	-.393	-1.299	.526	.406
N_LIMA1_WFL	-.148	-1.064	.773	.756
N_OCT4_ConA	-.210	-1.143	.729	.665
N_OCT4_AAL	-.226	-1.116	.671	.626
N_OCT4_UEA	-.665	-1.574	.262	.161
N_OCT4_MAA	.012	-.879	.902	.980
N_OCT4_SBA	-.427	-1.335	.493	.367
N_OCT4_WFL	.558	-.447	1.543	.281
N_MET_ConA	-.252	-1.185	.689	.604
N_MET_AAL	-.389	-1.284	.516	.403
N_MET_UEA	-.585	-1.488	.335	.120
N_MET_MAA	-.450	-1.347	.459	.336
N_MET_SBA	-.405	-1.312	.514	.392
N_MET_WFL	-.049	-.965	.869	.919
N_CIP2A_ConA	.565	-.353	1.467	.384
N_CIP2A_AAL	.077	-.815	.966	.868
N_CIP2A_UEA	-.376	-1.270	.529	.419
N_CIP2A_MAA	-.007	-.897	.884	.989
N_CIP2A_SBA	-.411	-1.318	.509	.386
N_CIP2A_WFL	-.195	-1.111	.727	.682
S_LIMA1_ConA	.440	-.485	1.354	.354
S_LIMA1_AAL	1.267	-.051	2.541	.060
S_LIMA1_UEA	-.299	-1.209	.618	.527
S_LIMA1_MAA	2.718	.819	4.538	.003
S_LIMA1_SBA	.184	-.729	1.092	.696
S_LIMA1_WFL	-.520	-1.437	.410	.276
S_OCT4_ConA	-.528	-1.771	.750	.428
S_OCT4_AAL	.301	-.985	1.558	.659
S_OCT4_UEA	-.580	-1.828	.705	.386
S_OCT4_MAA	-.367	-1.600	.891	.578
S_OCT4_SBA	-.319	-1.550	.934	.627
S_OCT4_WFL	-.281	-1.295	.746	.598
S_MET_ConA	.529	-.401	1.447	.268
S_MET_AAL	1.406	.064	2.700	.040
S_MET_UEA	.371	-.550	1.283	.434
S_MET_MAA	.212	-1.031	1.441	.745
S_MET_SBA	.160	-.752	1.069	.733
S_MET_WFL	-.125	-1.033	.786	.791
S_CIP2A_ConA	-.578	-1.497	.357	.228
S_CIP2A_AAL	.829	-.426	2.052	.516
S_CIP2A_UEA	-.227	-1.136	.687	.630
S_CIP2A_MAA	.411	-.852	1.646	.534
S_CIP2A_SBA	-.150	-1.058	.762	.750
S_CIP2A_WFL	-.273	-1.189	.651	.567

Diabetes mellitus typus 2

		95% Confidence Interval		
	γ (Hedges)	Lower	Upper	p
LIMA1_ConA	.287	-.564	1.132	.511
LIMA1_AAL	.453	-.368	1.265	.282
LIMA1_UEA	.303	-.517	1.117	.472
LIMA1_MAA	-.156	-.983	.674	.715
LIMA1_SBA	-.061	-.916	.796	.891
LIMA1_WFL	.052	-.865	.967	.913
OCT4_ConA	-.295	-1.141	.556	.500
OCT4_AAL	-.153	-.959	.656	.713
OCT4_UEA	-.416	-1.239	.417	.496
OCT4_MAA	-.453	-1.286	.390	.295
OCT4_SBA	-1.010	-1.917	-.082	.153
OCT4_WFL	-.806	-1.768	.179	.228
MET_ConA	.234	-.615	1.079	.592
MET_AAL	.194	-.616	1.001	.641
MET_UEA	.116	-.698	.928	.782
MET_MAA	-.234	-1.062	.598	.584
MET_SBA	-.916	-1.805	-.007	.180
MET_WFL	-.667	-1.600	.283	.304
CIP2A_ConA	-.510	-1.361	.352	.399
CIP2A_AAL	-.788	-1.616	.056	.244
CIP2A_UEA	.154	-.661	.966	.713
CIP2A_MAA	.037	-.790	.864	.930
CIP2A_SBA	-.654	-1.525	.232	.322
CIP2A_WFL	-1.080	-2.048	-.087	.180
N_LIMA1_ConA	.298	-.635	1.223	.535
N_LIMA1_AAL	.353	-.583	1.280	.464
N_LIMA1_UEA	-.061	-.985	.864	.898
N_LIMA1_MAA	.204	-.725	1.128	.670
N_LIMA1_SBA	.290	-.702	1.272	.571
N_LIMA1_WFL	-.175	-1.164	.820	.734
N_OCT4_ConA	.178	-.808	1.160	.727
N_OCT4_AAL	.343	-.593	1.269	.477
N_OCT4_UEA	.030	-.895	.953	.950
N_OCT4_MAA	-.181	-1.105	.747	.705
N_OCT4_SBA	-.046	-1.027	.937	.928
N_OCT4_WFL	-.758	-1.859	.370	.191
N_MET_ConA	.204	-.784	1.185	.690
N_MET_AAL	.336	-.599	1.262	.486
N_MET_UEA	.003	-.921	.927	.995
N_MET_MAA	-.129	-1.052	.798	.787
N_MET_SBA	-.475	-1.464	.527	.357
N_MET_WFL	-.566	-1.567	.453	.280
N_CIP2A_ConA	.310	-.624	1.235	.520
N_CIP2A_AAL	.145	-.782	1.068	.762
N_CIP2A_UEA	.022	-.902	.946	.963
N_CIP2A_MAA	-.245	-1.169	.686	.610
N_CIP2A_SBA	-.724	-1.726	.300	.168
N_CIP2A_WFL	-.841	-1.863	.206	.313
S_LIMA1_ConA	-.035	-.944	.874	.940
S_LIMA1_AAL	-.113	-1.197	.976	.842
S_LIMA1_UEA	-.676	-1.601	.266	.161
S_LIMA1_MAA	-.722	-2.142	.746	.633
S_LIMA1_SBA	.099	-.812	1.007	.834
S_LIMA1_WFL	-.143	-1.051	.769	.761
S_OCT4_ConA	.248	-1.162	1.641	.738
S_OCT4_AAL	-.049	-1.426	1.332	.947
S_OCT4_UEA	-.033	-1.428	1.364	.964
S_OCT4_MAA	-.147	-1.540	1.256	.842
S_OCT4_SBA	-.390	-1.787	1.033	.601
S_OCT4_WFL	.503	-.623	1.607	.230
S_MET_ConA	-.044	-.952	.866	.926
S_MET_AAL	-.113	-1.197	.975	.841
S_MET_UEA	-.213	-1.121	.701	.651
S_MET_MAA	-.609	-2.019	.842	.421
S_MET_SBA	-.103	-1.011	.808	.827
S_MET_WFL	-.118	-1.026	.794	.803
S_CIP2A_ConA	-.044	-.953	.865	.925
S_CIP2A_AAL	-.061	-1.145	1.026	.914
S_CIP2A_UEA	.036	-.873	.944	.939
S_CIP2A_MAA	-1.052	-2.517	.477	.183
S_CIP2A_SBA	.148	-.764	1.056	.754
S_CIP2A_WFL	.339	-.588	1.257	.477

Chronic pulmonary disease (COPD, asthma)

		95% Confidence Interval		
	γ (Hedges)	Lower	Upper	p
LIMA1_ConA	.620	-.436	1.663	.252
LIMA1_AAL	.562	-.401	1.514	.255
LIMA1_UEA	.068	-.881	1.015	.889
LIMA1_MAA	-.459	-1.418	.511	.357
LIMA1_SBA	.063	-.977	1.101	.907
LIMA1_WFL	-.134	-1.183	.918	.805
OCT4_ConA	.605	-.450	1.647	.264
OCT4_AAL	.446	-.511	1.395	.363
OCT4_UEA	-.842	-1.816	.150	.425
OCT4_MAA	-.505	-1.466	.468	.546
OCT4_SBA	-.620	-1.672	.447	.560
OCT4_WFL	-.251	-1.309	.815	.648
MET_ConA	.594	-.460	1.635	.272
MET_AAL	.566	-.398	1.518	.252
MET_UEA	.005	-.942	.953	.991
MET_MAA	-.261	-1.216	.701	.598
MET_SBA	-.551	-1.596	.508	.596
MET_WFL	-.014	-1.063	1.035	.979
CIP2A_ConA	.369	-.674	1.405	.491
CIP2A_AAL	-.147	-1.091	.800	.763
CIP2A_UEA	-.030	-.977	.918	.951
CIP2A_MAA	.054	-.902	1.009	.913
CIP2A_SBA	-.476	-1.520	.578	.614
CIP2A_WFL	.227	-.828	1.276	.677
N_LIMA1_ConA	-.156	-1.332	1.025	.799
N_LIMA1_AAL	.275	-.910	1.452	.653
N_LIMA1_UEA	.112	-1.068	1.288	.855
N_LIMA1_MAA	-.001	-1.179	1.176	.998
N_LIMA1_SBA	.224	-.962	1.404	.715
N_LIMA1_WFL	.202	-.987	1.384	.742
N_OCT4_ConA	-.446	-1.847	.969	.541
N_OCT4_AAL	.340	-.848	1.518	.579
N_OCT4_UEA	-1.613	-2.888	-.299	.016
N_OCT4_MAA	-.931	-2.136	.300	.140
N_OCT4_SBA	-.537	-1.724	.666	.386
N_OCT4_WFL	-.672	-1.878	.558	.288
N_MET_ConA	-.232	-1.630	1.174	.750
N_MET_AAL	.302	-.885	1.479	.622
N_MET_UEA	-.410	-1.590	.782	.505
N_MET_MAA	.243	-.941	1.420	.691
N_MET_SBA	.200	-.986	1.379	.745
N_MET_WFL	-.153	-1.335	1.034	.804
N_CIP2A_ConA	.161	-1.020	1.338	.791
N_CIP2A_AAL	-.174	-1.351	1.007	.775
N_CIP2A_UEA	-2.790	-4.266	-1.264	.134
N_CIP2A_MAA	-.839	-2.038	.383	.181
N_CIP2A_SBA	-1.789	-3.098	-.436	.237
N_CIP2A_WFL	.036	-1.148	1.219	.953
S_LIMA1_ConA	.149	-.900	1.193	.784
S_LIMA1_AAL	-.136	-1.535	1.269	.853
S_LIMA1_UEA	-.111	-1.156	.936	.837
S_LIMA1_MAA	-.392	-2.236	1.478	.689
S_LIMA1_SBA	-.105	-1.149	.943	.846
S_LIMA1_WFL	-.170	-1.214	.880	.754
S_OCT4_ConA	.511	-1.372	2.358	.605
S_OCT4_AAL	.378	-1.434	2.155	.694
S_OCT4_UEA	.380	-1.490	2.224	.699
S_OCT4_MAA	-.303	-2.145	1.561	.757
S_OCT4_SBA	-.785	-2.649	1.131	.432
S_OCT4_WFL	-.002	-1.201	1.198	.998
S_MET_ConA	.300	-.754	1.346	.580
S_MET_AAL	-.268	-1.668	1.143	.714
S_MET_UEA	-.589	-1.643	.480	.283
S_MET_MAA	.283	-1.579	2.125	.773
S_MET_SBA	.114	-.934	1.159	.833
S_MET_WFL	-.139	-1.183	.910	.798
S_CIP2A_ConA	.001	-1.045	1.046	.999
S_CIP2A_AAL	.002	-1.399	1.403	.998
S_CIP2A_UEA	-.515	-1.566	.550	.347
S_CIP2A_MAA	-1.200	-3.109	.784	.243
S_CIP2A_SBA	.198	-.852	1.242	.930
S_CIP2A_WFL	-.048	-1.097	1.002	.930

Sex

		95% Confidence Interval		
	γ (Hedges)	Lower	Upper	p
LIMA1_ConA	-.413	-1.193	.376	.307
LIMA1_AAL	-.667	-1.447	.128	.147
LIMA1_UEA	-.872	-1.688	-.039	.099
LIMA1_MAA	-.514	-1.358	.343	.335
LIMA1_SBA	-.447	-1.260	.377	.290
LIMA1_WFL	-.924	-1.820	-.005	.118
OCT4_ConA	.036	-.740	.811	.929
OCT4_AAL	-.117	-.880	.649	.767
OCT4_UEA	-.519	-1.335	.309	.343
OCT4_MAA	-.173	-1.008	.667	.690
OCT4_SBA	-.139	-.973	.700	.749
OCT4_WFL	-.234	-1.167	.706	.629
MET_ConA	-.435	-1.215	.356	.284
MET_AAL	-.742	-1.527	.058	.069
MET_UEA	-.539	-1.333	.266	.282
MET_MAA	-.081	-.916	.756	.852
MET_SBA	-.151	-.957	.660	.718
MET_WFL	-.253	-1.110	.611	.570
CIP2A_ConA	-.037	-.812	.738	.926
CIP2A_AAL	.027	-.737	.790	.945
CIP2A_UEA	-.724	-1.528	.096	.156
CIP2A_MAA	-.305	-1.142	.540	.483
CIP2A_SBA	-.205	-1.012	.607	.624
CIP2A_WFL	.270	-.595	1.127	.545
N_LIMA1_ConA	.301	-.600	1.192	.517
N_LIMA1_AAL	.317	-.585	1.209	.495
N_LIMA1_UEA	.528	-.387	1.429	.261
N_LIMA1_MAA	.357	-.546	1.250	.443
N_LIMA1_SBA	.329	-.586	1.233	.485
N_LIMA1_WFL	.637	-.344	1.598	.206
N_OCT4_ConA	.342	-.573	1.247	.468
N_OCT4_AAL	.261	-.638	1.151	.574
N_OCT4_UEA	.521	-.394	1.421	.267
N_OCT4_MAA	.254	-.644	1.145	.583
N_OCT4_SBA	.224	-.685	1.126	.633
N_OCT4_WFL	.197	-.782	1.168	.698
N_MET_ConA	.419	-.501	1.327	.376
N_MET_AAL	.393	-.512	1.287	.399
N_MET_UEA	.602	-.319	1.506	.109
N_MET_MAA	.569	-.349	1.471	.227
N_MET_SBA	.417	-.503	1.324	.379
N_MET_WFL	.686	-.299	1.651	.175
N_CIP2A_ConA	.408	-.499	1.302	.382
N_CIP2A_AAL	.318	-.583	1.210	.493
N_CIP2A_UEA	.366	-.538	1.260	.431
N_CIP2A_MAA	.011	-.879	.901	.980
N_CIP2A_SBA	.047	-.856	.949	.920
N_CIP2A_WFL	.489	-.478	1.441	.326
S_LIMA1_ConA	-.042	-.885	.803	.924
S_LIMA1_AAL	.170	-.826	1.160	.742
S_LIMA1_UEA	-.202	-1.045	.647	.645
S_LIMA1_MAA	-.371	-1.542	.825	.553
S_LIMA1_SBA	.007	-.837	.850	.987
S_LIMA1_WFL	-.365	-1.212	.491	.407
S_OCT4_ConA	-.241	-1.407	.941	.698
S_OCT4_AAL	-1.279	-2.711	.240	.103
S_OCT4_UEA	-.572	-1.757	.651	.453
S_OCT4_MAA	-.394	-1.565	.805	.530
S_OCT4_SBA	-.591	-1.778	.635	.354
S_OCT4_WFL	-.579	-1.701	.570	.330
S_MET_ConA	-.133	-.976	.714	.761
S_MET_AAL	.030	-.962	1.020	.954
S_MET_UEA	.292	-.561	1.137	.507
S_MET_MAA	-1.182	-2.466	.167	.166
S_MET_SBA	.002	-.841	.846	.996
S_MET_WFL	-.164	-1.007	.684	.708
S_CIP2A_ConA	-.433	-1.282	.428	.328
S_CIP2A_AAL	-.104	-1.094	.889	.840
S_CIP2A_UEA	.248	-.603	1.092	.572
S_CIP2A_MAA	-.083	-1.249	1.089	.893
S_CIP2A_SBA	-.412	-1.260	.448	.351
S_CIP2A_WFL	-.010	-.870	.851	.983

Age

		95% Confidence Intervals		
	tau_b (Kendall's)	Lower	Upper	p
LIMA1_ConA	.322	.044	.553	.030
LIMA1_AAL	.268	-.008	.506	.064
LIMA1_UEA	.022	-.261	.302	.881
LIMA1_MAA	.154	-.149	.431	.322
LIMA1_SBA	.165	-.130	.433	.277
LIMA1_WFL	.167	-.154	.456	.312
OCT4_ConA	.129	-.158	.397	.383
OCT4_AAL	.071	-.208	.340	.623
OCT4_UEA	-.020	-.307	.270	.895
OCT4_MAA	-.057	-.348	.243	.713
OCT4_SBA	-.040	-.332	.260	.799
OCT4_WFL	-.047	-.374	.291	.790
MET_ConA	.226	-.060	.477	.129
MET_AAL	.160	-.121	.417	.271
MET_UEA	.030	-.254	.309	.842
MET_MAA	.013	-.284	.308	.932
MET_SBA	.085	-.209	.365	.578
MET_WFL	-.059	-.366	.259	.720
CIP2A_ConA	.085	-.202	.358	.567
CIP2A_AAL	.119	-.162	.382	.412
CIP2A_UEA	.081	-.205	.355	.584
CIP2A_MAA	.172	-.131	.445	.270
CIP2A_SBA	.206	-.088	.467	.176
CIP2A_WFL	.005	-.308	.318	.974
N_LIMA1_ConA	-.059	-.375	.268	.726
N_LIMA1_AAL	.166	-.165	.464	.326
N_LIMA1_UEA	-.119	-.425	.212	.483
N_LIMA1_MAA	.119	-.212	.425	.483
N_LIMA1_SBA	.020	-.315	.351	.909
N_LIMA1_WFL	.067	-.284	.402	.710
N_OCT4_ConA	-.013	-.345	.321	.939
N_OCT4_AAL	-.083	-.395	.246	.623
N_OCT4_UEA	.024	-.301	.344	.888
N_OCT4_MAA	-.024	-.344	.301	.888
N_OCT4_SBA	-.166	-.473	.177	.342
N_OCT4_WFL	-.271	-.584	.113	.164
N_MET_ConA	-.080	-.402	.260	.648
N_MET_AAL	.036	-.290	.354	.833
N_MET_UEA	.190	-.141	.483	.261
N_MET_MAA	.154	-.177	.454	.362
N_MET_SBA	-.099	-.419	.242	.569
N_MET_WFL	-.067	-.402	.284	.710
N_CIP2A_ConA	-.190	-.483	.141	.261
N_CIP2A_AAL	.036	-.290	.354	.833
N_CIP2A_UEA	-.012	-.333	.312	.944
N_CIP2A_MAA	.202	-.129	.492	.233
N_CIP2A_SBA	-.106	-.424	.235	.543
N_CIP2A_WFL	-.126	-.451	.228	.483
S_LIMA1_ConA	-.215	-.495	.105	.192
S_LIMA1_AAL	-.101	-.471	.299	.620
S_LIMA1_UEA	.258	-.060	.528	.118
S_LIMA1_MAA	.254	-.310	.685	.345
S_LIMA1_SBA	.054	-.264	.361	.744
S_LIMA1_WFL	.215	-.105	.495	.192
S_OCT4_ConA	.254	-.310	.685	.345
S_OCT4_AAL	.429	-.282	.836	.176
S_OCT4_UEA	-.028	-.542	.501	.917
S_OCT4_MAA	.085	-.458	.581	.753
S_OCT4_SBA	.028	-.501	.542	.917
S_OCT4_WFL	.062	-.377	.477	.783
S_MET_ConA	-.172	-.460	.149	.297
S_MET_AAL	-.034	-.417	.359	.869
S_MET_UEA	.215	-.105	.495	.192
S_MET_MAA	.254	-.310	.685	.345
S_MET_SBA	-.065	-.370	.254	.696
S_MET_WFL	.204	-.116	.486	.216
S_CIP2A_ConA	.000	-.313	.313	1.000
S_CIP2A_AAL	-.034	-.417	.359	.869
S_CIP2A_UEA	.290	-.025	.553	.078
S_CIP2A_MAA	.085	-.458	.581	.753
S_CIP2A_SBA	-.108	-.407	.213	.514
S_CIP2A_WFL	.102	-.228	.411	.550
