# Supplementary material for: Identification of stemness-related glycosylation changes in head and neck squamous cell carcinoma
Source: BMC Cancer. 2024 Apr 10;24:443. doi: 10.1186/s12885-024-12161-5 (PMC11005150; doi:10.1186/s12885-024-12161-5)
Supplement: Supplementary file 5 — Supplementary Material 5 [file 12885_2024_12161_MOESM5_ESM.pdf]

Supplemental Table 4. **Correlation between tumor tissue, adjacent (normal) tissue and serum**

LIMA1		Correlation Kendall's tau_b	95% CI Lower	Upper	P
**ConA**	LIMA1_ConA - N_LIMA1_ConA	-.020	-.350	.316	.910
	LIMA1_ConA - S_LIMA1_ConA	-.276	-.535	.031	.080
	N_LIMA1_ConA - S_LIMA1_ConA	-.133	-.469	.235	.471
**AAL**	LIMA1_AAL - N_LIMA1_AAL	.064	-.264	.379	.700
	LIMA1_AAL - S_LIMA1_AAL	-.077	-.452	.321	.702
	N_LIMA1_AAL - S_LIMA1_AAL	.244	-.272	.652	.325
**UEA**	LIMA1_UEA - N_LIMA1_UEA	.190	-.153	.492	.272
	LIMA1_UEA - S_LIMA1_UEA	-.147	-.440	.174	.364
	N_LIMA1_UEA - S_LIMA1_UEA	.050	-.313	.400	.787
**MAA**	LIMA1_MAA - N_LIMA1_MAA	-.059	-.395	.292	.742
	LIMA1_MAA - S_LIMA1_MAA	.071	-.520	.617	.805
	N_LIMA1_MAA - S_LIMA1_MAA	.200	-.613	.807	.573
**SBA**	LIMA1_SBA - N_LIMA1_SBA	-.167	-.495	.203	.368
	LIMA1_SBA - S_LIMA1_SBA	-.255	-.542	.085	.140
	N_LIMA1_SBA - S_LIMA1_SBA	.181	-.205	.518	.347
**WFL**	LIMA1_WFL - N_LIMA1_WFL	.205	-.220	.565	.329
	LIMA1_WFL - S_LIMA1_WFL	.181	-.205	.518	.347
	N_LIMA1_WFL - S_LIMA1_WFL	-.033	-.416	.360	.870

OCT4		Correlation Kendall's tau_b	95% CI Lower	Upper	P
**ConA**	OCT4_ConA - N_OCT4_ConA	-.046	-.373	.292	.791
	OCT4_ConA - S_OCT4_ConA	-.056	-.562	.481	.835
	N_OCT4_ConA - S_OCT4_ConA	.467	-.389	.890	.188
**AAL**	OCT4_AAL - N_OCT4_AAL	.181	-.150	.476	.278
	OCT4_AAL - S_OCT4_AAL	-.048	-.662	.605	.881
	N_OCT4_AAL - S_OCT4_AAL	.200	-.798	.905	.624
**UEA**	OCT4_UEA - N_OCT4_UEA	.255	-.085	.542	.140
	OCT4_UEA - S_OCT4_UEA	-.389	-.757	.167	.144
	N_OCT4_UEA - S_OCT4_UEA	-.333	-.852	.515	.348
**MAA**	OCT4_MAA - N_OCT4_MAA	.176	-.179	.491	.323
	OCT4_MAA - S_OCT4_MAA	-.214	-.699	.405	.458
	N_OCT4_MAA - S_OCT4_MAA	.467	-.389	.890	.188
**SBA**	OCT4_SBA - N_OCT4_SBA	.033	-.328	.386	.857
	OCT4_SBA - S_OCT4_SBA	-.143	-.659	.465	.621
	N_OCT4_SBA - S_OCT4_SBA	.400	-.702	.938	.327
**WFL**	OCT4_WFL - N_OCT4_WFL	.051	-.363	.449	.807
	OCT4_WFL - S_OCT4_WFL	.071	-.520	.617	.805
	N_OCT4_WFL - S_OCT4_WFL	-.467	-.890	.389	.188

MET		Correlation Kendall's tau_b	95% CI Lower	Upper	P
**ConA**	MET_ConA - N_MET_ConA	-.072	-.395	.268	.677
	MET_ConA - S_MET_ConA	-.276	-.535	.031	.080
	N_MET_ConA - S_MET_ConA	.033	-.328	.386	.857
**AAL**	MET_AAL - N_MET_AAL	-.006	-.328	.317	.972
	MET_AAL - S_MET_AAL	-.165	-.520	.239	.412
	N_MET_AAL - S_MET_AAL	.378	-.131	.729	.128
**UEA**	MET_UEA - N_MET_UEA	.216	-.126	.512	.211
	MET_UEA - S_MET_UEA	-.147	-.440	.174	.364
	N_MET_UEA - S_MET_UEA	.167	-.203	.495	.368
**MAA**	MET_MAA - N_MET_MAA	-.162	-.480	.194	.365
	MET_MAA - S_MET_MAA	.071	-.520	.617	.805
	N_MET_MAA - S_MET_MAA	.200	-.613	.807	.573
**SBA**	MET_SBA - N_MET_SBA	.017	-.343	.372	.928
	MET_SBA - S_MET_SBA	-.229	-.522	.113	.185
	N_MET_SBA - S_MET_SBA	.352	-.022	.640	.067
**WFL**	MET_WFL - N_MET_WFL	.103	-.318	.489	.625
	MET_WFL - S_MET_WFL	-.086	-.443	.296	.656
	N_MET_WFL - S_MET_WFL	.253	-.150	.584	.208

CIP2A		Correlation Kendall's tau_b	95% CI Lower	Upper	P
**ConA**	CIP2A_ConA - N_CIP2A_ConA	.203	-.140	.502	.240
	CIP2A_ConA - S_CIP2A_ConA	.019	-.287	.321	.904
	N_CIP2A_ConA - S_CIP2A_ConA	.183	-.186	.508	.322
**AAL**	CIP2A_AAL - N_CIP2A_AAL	.018	-.307	.338	.916
	CIP2A_AAL - S_CIP2A_AAL	.099	-.301	.469	.622
	N_CIP2A_AAL - S_CIP2A_AAL	.289	-.228	.678	.245
**UEA**	CIP2A_UEA - N_CIP2A_UEA	.059	-.280	.384	.733
	CIP2A_UEA - S_CIP2A_UEA	.074	-.245	.378	.650
	N_CIP2A_UEA - S_CIP2A_UEA	.033	-.328	.386	.857
**MAA**	CIP2A_MAA - N_CIP2A_MAA	-.309	-.591	.040	.084
	CIP2A_MAA - S_CIP2A_MAA	-.214	-.699	.405	.458
	N_CIP2A_MAA - S_CIP2A_MAA	.200	-.613	.807	.573
**SBA**	CIP2A_SBA - N_CIP2A_SBA	-.283	-.582	.083	.126
	CIP2A_SBA - S_CIP2A_SBA	-.203	-.502	.140	.240
	N_CIP2A_SBA - S_CIP2A_SBA	.172	-.213	.511	.372
**WFL**	CIP2A_WFL - N_CIP2A_WFL	.179	-.245	.546	.393
	CIP2A_WFL - S_CIP2A_WFL	.165	-.239	.520	.412
	N_CIP2A_WFL - S_CIP2A_WFL	.282	-.141	.618	.180
